# Study on the influence of TiO_2_ nanoparticles on the breakdown voltage of transformer oil under severe cold conditions

**DOI:** 10.1371/journal.pone.0307230

**Published:** 2025-01-09

**Authors:** Chunxu Qin, Wenjie Lin, Yongxiang Huang, Liqiang Liu, Huichun Hua, Huijuan Liang

**Affiliations:** 1 College of Electric Power, Inner Mongolia University of Technology, Hohhot, China; 2 Engineering Research Center of Large Energy Storage Technology, Ministry of Education, Hohhot, China; 3 Xinyang Power Supply Co, State Grid Henan Prov Power Co, Xinyang, China; 4 State Key Laboratory of Alternate Electrical Power System with Renewable Energy Sources (North China Electric Power University), Hebei Province, Baoding, China; indian institute of technology jammu, INDIA

## Abstract

The modified nanoparticles can significantly improve the insulation characteristics of transformer oil. Currently, there is a lack of research on the actual motion state of particles in nanofluid to further understand the micro-mechanism of nanoparticles improving the insulation characteristics of transformer oil. In this study, the nanofluid containing 0.01g/L of TiO_2_ with a particle size of 20nm is prepared using the thermal oscillation method. Breakdown voltage tests are carried out. The experimental test results show that adding nanoparticles can significantly reduce the breakdown probability of transformer oil. The more the water content, the less the enhancement effect of the nanofluid on breakdown voltage. The higher the temperature, the stronger the enhancement effect of the nanofluid on breakdown voltage. Finally, the polarization process of nanoparticles and the trajectory of charged particles in the transformer oil under different electric fields are simulated using COMSOL to further analyze the influence mechanism of nanoparticles on the insulation characteristics of transformer oil. The simulation results show that under the action of the electric field, nanoparticles polarize and generate charge shallow traps to adsorb electrons, reducing the high-speed free charges in the oil, and indirectly increasing the breakdown voltage.

## Introduction

Transformer oil is an important insulation and cooling medium for electrical equipment, and its performance directly affects the stable operation of electrical equipment. Under the influence of harsh environmental factors in northern China during winter, the performance of transformer oil can be greatly affected. The most obvious consequence is a decrease in breakdown voltage, leading to transformer failures that can threaten the reliable operation of substations and the entire power system [1, 2]. Therefore, it is of great significance to improve the performance of transformer oil to enhance the stable operation of transformers and power systems [3–6].

Current research suggests that the addition of nanoparticles with appropriate particle sizes to mineral oils can moderately enhance various physical and chemical properties of the oils. nanoparticles can improve transformer oil’s physical properties to some extent [7, 8]. Adding oxidized multi-walled carbon nanotubes (MWCNTs) to transformer oil can increase the thermal conductivity of nanofluids and transformer oil with the increase in temperature, and the viscosity of nanofluids is lower than that of transformer oil [9]. Then it was added to the transformer oil, and it was found that the thermal conductivity and flash point of the nanofluid increased with the increase of temperature. The maximum enhancement in the VI of nanofluids at 0.2 wt% of hybrid nanoparticles was 34.86%. The maximum improvement in natural and forced heat transfer coefficient of nanofluids (NCHT and FCHT), increased by 23.08% and 24.68%, respectively [10]. The ferromagnetic MWNT-based water nanofluids were prepared by stably dispersing MWNT in an aqueous medium. It was found that the rheological and thermo-physical properties of ferromagnetic samples were significantly compared to the covalent nanofluids and certainly pure water. Maximum thermal conductivity,0.897 W/m K, was obtained for the MWNT-Fe-based water nanofluid at a concentration of 0.2 wt% and temperature of 80°C [11]. Covalently functionalized-hydroxylated multi-walled carbon nanotubes (MWCNTs-OH) have similar properties [12].

In terms of breakdown voltage, nanoparticles have varying degrees of enhancement [13–15]. Researchers both domestically and internationally have analyzed different types of nanoparticles and classified them into three categories: conductive nanoparticles such as Fe_2_O_3_, Fe_3_O_4_, SiC, dielectric nanoparticles such as Al_2_O_3_, SiO_2_, AlN, and semiconductor nanoparticles such as TiO_2_ [16]. Comparative experiments on lightning impulse breakdown of nanofluid with Fe_3_O_4_ nanoparticles added showed that the positive polarity breakdown voltage of transformer oil significantly increased by over 90%, and the propagation speed of positive polarity streamer discharge decreased by 46% due to the presence of nanoparticles [17]. For SiO_2_ particles, the discharge mechanism of SiO_2_ nanofluid under DC voltage depends on the polarity: under negative polarity, the partial discharge inception voltage and total discharge intensity of nanofluid are essentially the same as mineral oil; while under positive polarity, the partial discharge inception voltage of nanofluid is increased compared to mineral oil, and the total discharge intensity is decreased [18]. Under approximate load conditions and operating conditions, the addition of aluminum nitride nanoceramic particles can reduce the temperature of transformer oil and the temperature near the winding hot spot, thereby improving the heat dissipation efficiency of the transformer [19]. Nanoparticles enhance the insulation characteristics of transformer oil and contribute to the increased breakdown voltage of oil-paper insulation. The nanofluid-paper system with a 5mm gap exhibits a 7.3% increase in breakdown voltage compared to the oil-paper system [20]. Compared to ordinary transformer oil, Fe_3_O_4_, TiO_2_, and Al_2_O_3_ nanofluid exhibit a 20.6%, 26.8%, and 27.6% increase, respectively, in the positive polarity lightning impulse breakdown voltage compared to pure transformer oil. The optimal mass concentrations for the three types of nanoparticles are 0.03 g/L, 0.01 g/L, and 0.02 g/L, respectively. It can be observed that TiO_2_ requires the lowest concentration to achieve the highest breakdown voltage [21].

Different nanoparticles exhibit varying degrees of improvement in the breakdown voltage of oil samples, with TiO_2_ and SiO_2_ nanoparticles showing the most significant enhancement [22, 23]. For TiO_2_ nanofluid, the highest increase in breakdown voltage is achieved at a nanoparticle concentration of 0.01g/L [24].

TiO_2_ nanofluid, when compared to pure oil, demonstrates a 23.8% increase in negative polarity breakdown voltage and a 6.3% increase in positive polarity breakdown voltage. Nanofluid suppresses the intensity of negative polarity corona discharge, and a significant decrease in the area of corona-induced luminescence, as well as the frequency and amplitude of light pulses and current pulses [25]. TiO_2_ nanoparticles can alter the ion migration rate in transformer oil. Nanosized TiO_2_ particles bind with water molecules at their surfaces to form aqueous films, and larger TiO_2_ nanoparticles exhibit significant viscous resistance when interacting with oils. When the applied field strength is low, TiO_2_ nanoparticles are relatively easier to transport due to the small viscous resistance, and instead become the main charge carriers, resulting in the ion mobility of TiO_2_ nano-modified oil under low field conditions higher than that in pure oil. As the intensity of the electric field progressively increases, the TiO_2_ nanoparticles are subjected to greater electrostatic forces, which gradually overcome the viscous resistance. Under low electric field conditions, the ion migration rate in nanofluid is 5.8 times higher than that in pure oil. However, under a high electric field, the ion mobility in nanofluid is only half of that in pure oil, so the breakdown voltage of nanofluid increases [26]. In needle-plate electrode experiments, it was found that TiO_2_-modified nanofluid significantly increases the flashover voltage of oil-impregnated paperboard and prolongs the flashover interruption time. Additionally, the modification effect of nanofluid on oil-paper insulation gradually strengthens as the nanoparticle size decreases. Compared to pure oil, when the nanoparticle size is 5 nm, the flashover voltage of the nanofluid increases by 35.8%, which is nearly 13.5% higher than that of the 10 nm and 15 nm cases [27].

**Fig 1:**
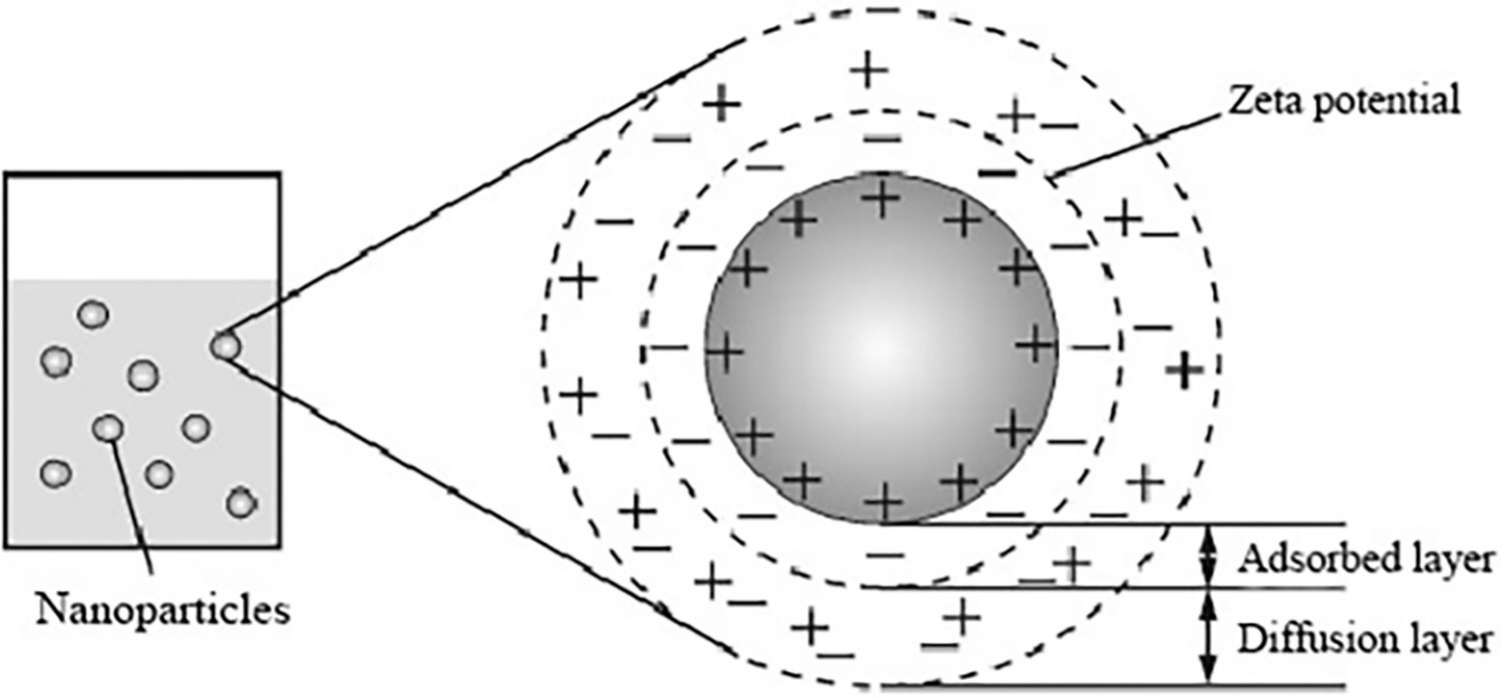
Stern double layer model.

For stable TiO_2_ nanofluid fluid, the nanoparticles disperse uniformly within the solution, forming a colloid. According to colloid chemistry theory, charge separation occurs at the interface between the solid and liquid phases. When solid particles come into contact with the solution, they adsorb charges on their surfaces, attracting opposite charges to surround them. The negative charges tightly bind to the surface of the nanoparticles, forming an adsorption layer (which contains deep traps), while positive charges and unadsorbed negative charges diffuse into the oil outside the adsorption layer, constituting a diffused layer of the double-layer structure, as shown in Fig 1 [28]. When electrons reach the diffusion layer, due to their same electrical charge, they are repelled by the nanoparticles via electrostatic force and change direction, making them less likely to collide with water molecules adsorbed on the surface of the nanoparticles. The streamer channels produced by electron collisions with nanoparticles are difficult to form through a continuous path. Therefore, the addition of nanoparticles to transformer oil is beneficial for increasing the breakdown voltage [29].

Currently, there are several models for enhancing the breakdown voltage of transformer oil with nanoparticles, including the shallow trap model, the potential trap model, and the electron capture model. The shallow trap model is the most widely applied [30]. Simulations of the motion of nanoparticles and charged particles in oil can further elucidate their mechanisms. In reference [24], the motion of nanoparticles in oil was simulated using a charge-carrying method, without considering the influence of dielectrophoretic forces, which does not align with reality.

Low temperatures can worsen the flow characteristics of transformer oil and alter the distribution of water in the oil, thereby affecting its insulation performance. Furthermore, cold starts of oil-immersed equipment, such as transformers, may result in potential failures [31, 32]. When the temperature is low, water in transformer oil can form ice crystals. The changes in the state of water and other impurities at low temperatures directly impact the insulation properties of transformer oil [33, 34].

To investigate the influence of TiO_2_ nanoparticles on the breakdown voltage of transformer oil under extreme cold conditions, this study uses Xinjiang Karamay #45 transformer oil as the base fluid and mixes it with prepared TiO_2_ nanoparticles to create nanomodified oil. Comparative experiments of breakdown tests is are conducted between the prepared nanofluid and pure oil, considering various influencing factors including various water contents, temperatures, the breakdown tests of pure oil and nanofluid were carried out at different temperatures, different water contents, and different, and electric fields strengths. Additionally, COMSOL Multiphysics 5.6 was used to simulate the velocity and trajectory of TiO_2_ nanoparticles under different electric field intensities. Based on simulation, the influence of polarized nanoparticles on the movement of charged particles in oil is discussed, and the influence mechanism of nanoparticles in oil on the breakdown voltage of oil gap is further analyzed.

## Experiment

### Sample preparation

#### Preparation of the TiO_2_nanomodified transformer oil sample

#45 transformer oil is chosen as the base fluid and subjected to filtration and drying treatment. For TiO_2_ nanoparticles, unprocessed nanoparticles with a white powder appearance and an average particle size of approximately 20 nm are selected.

**Fig 2:**
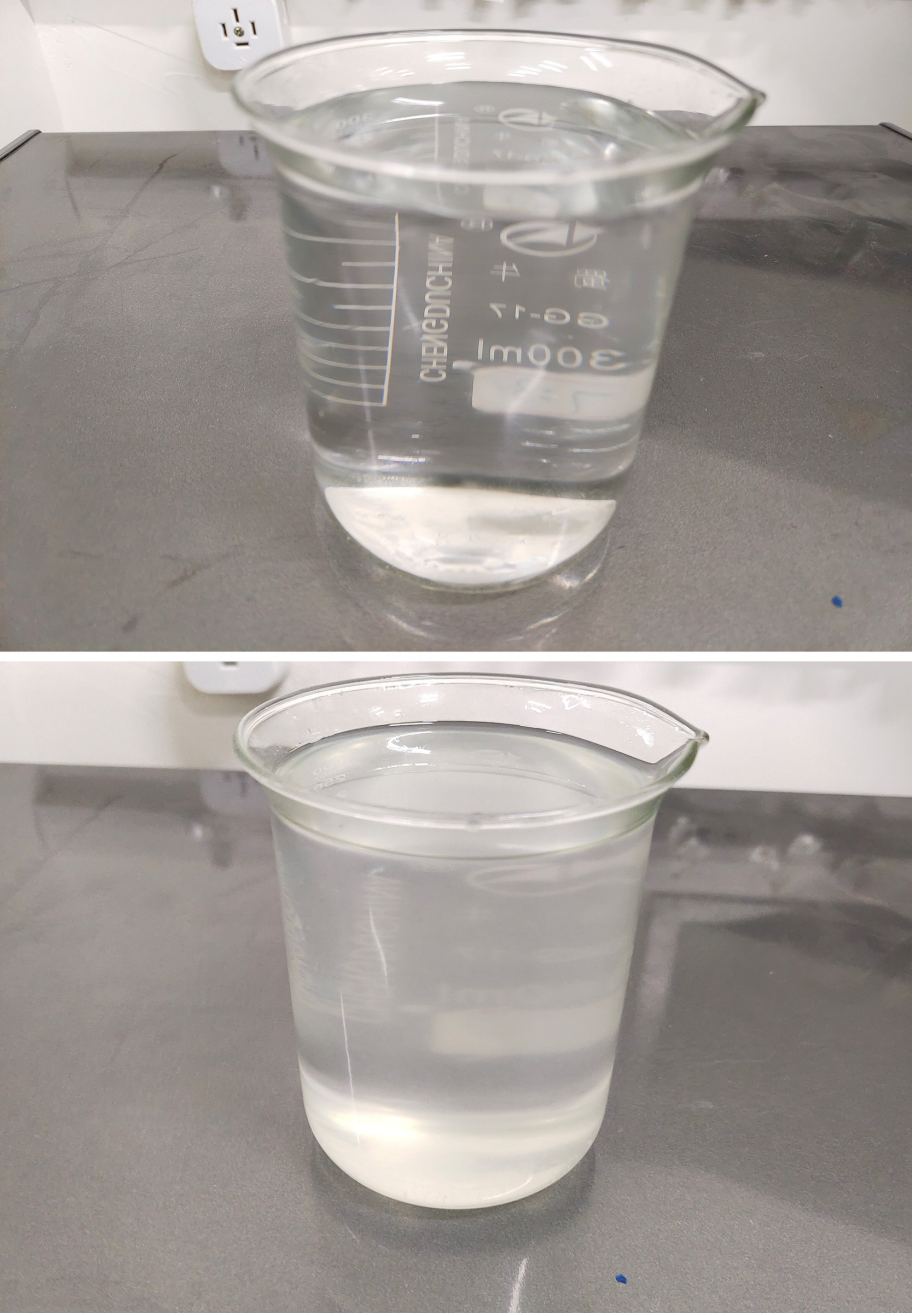
Oil samples used in the test. (a) Pure oil, (b) 0.01g/L Nanofluid.

To obtain a stable nanofluid, the nanoparticles are first dissolved in a portion of the base fluid during the preparation process. After thorough dispersion, the solution is mixed with the remaining base fluid for a secondary dispersion. The environmental conditions during the operation are room temperature (20°C) and a humidity of 32% RH. The specific procedure is as follows:

1. An appropriate amount of TiO_2_ powder was accurately weighed and placed into a beaker. A minor quantity of pure #45 transformer oil was then poured into the beaker, and a glass rod was used to stir the mixture, ensuring that the nanoparticles were thoroughly dispersed within the transformer oil. Following this, the mixture was placed in an electrically heated constant-temperature cabinet and heated for more than 3 hours, with stirring occurring at intervals of every 15 minutes to promote a homogenous blend of nanoparticles and oil.
2. The obtained oil sample was transferred into a glass syringe, which was then placed in a fully automatic oscillator. Each cycle of the process involved heating for 10 minutes followed by 20 minutes of shaking, after repeating this cycle twice, yielding the nanofluid sample.
3. The nanofluid was mixed with the remaining transformer oil and stirred before being transferred back into the syringe. The procedure outlined in section (2) was then repeated to obtain a stable nanofluid. After being left to settle for a day, no significant precipitation was observed.
4. Place the prepared nanofluid sample in an electrically heated drying oven and allow it to stand still and dry for more than 72 hours to evaporate as much water as possible. Using the method above, prepare nanofluid samples with a concentration of 0.01 g/L. The oil sample is shown in Fig 2.

The specifications of the #45 transformer oil and TiO_2_ nanoparticles are presented in Tables 1 and 2, respectively.

**Table 1:** Parametric specifications of #45 transformer oil.

Parameter	#45 transformer oil
**Relative dielectric constant**	2.2
**Electric conductivity / [S/m]**	1×10^−14^
**Density (20°C)/ [kg/m** ^**3**^ **]**	880
**Specific heat capacity (20°C)/ [J/(kg∙K)]**	1700
**Thermal conductivity /[W/(m∙K)]**	0.13
**Flashpoint /[°C]**	147
**Viscosity (40°C)/ [cSt]**	9.8

**Table 2:** Parametric specifications of TiO_2_ nanoparticles.

Parameter	TiO_2_
**Relative dielectric constant**	114
**Electric conductivity / [S/m]**	1×10^−11^
**Density (20°C)/ [kg/m** ^**3**^ **]**	3900

**Fig 3:**
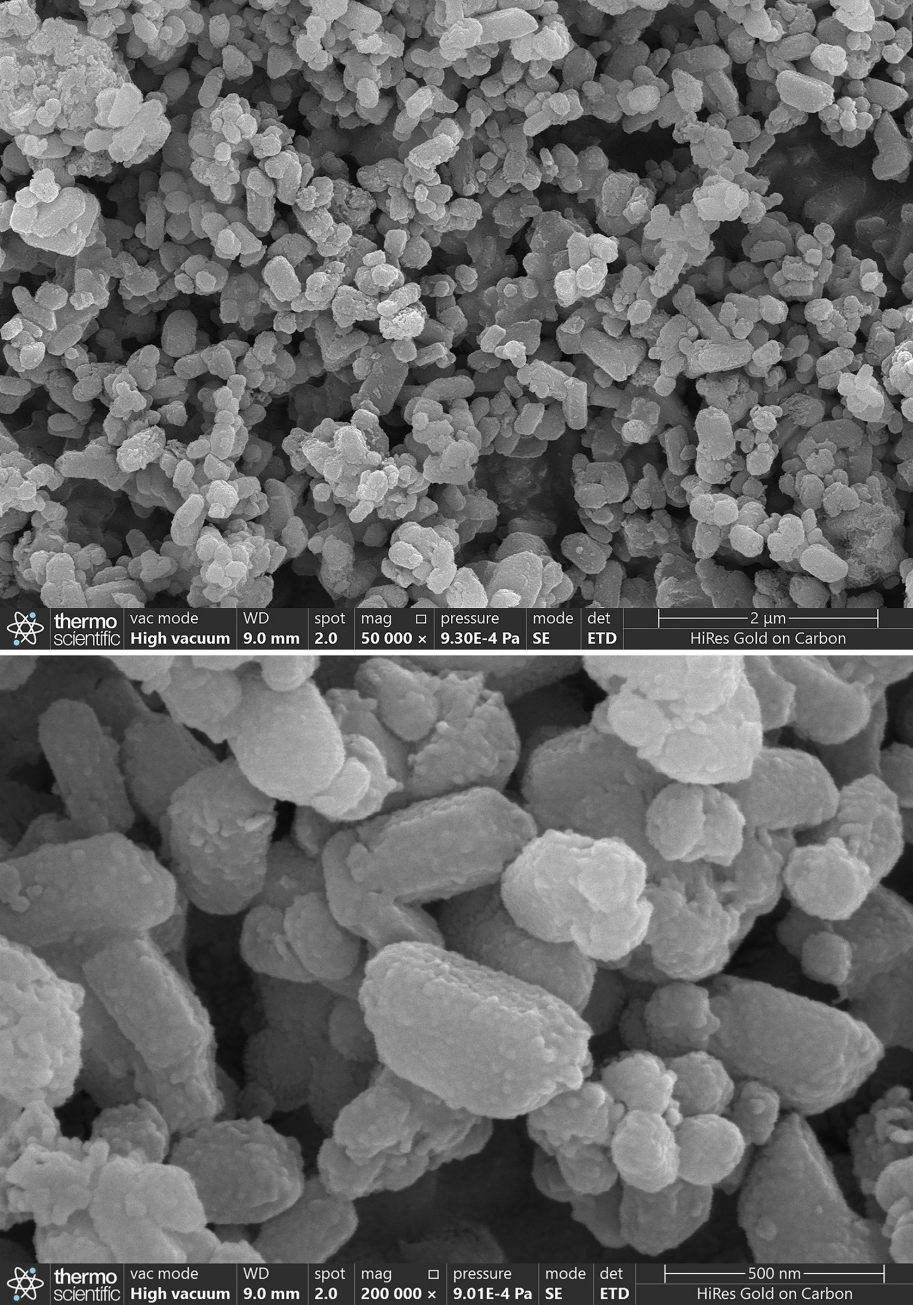
SEM analyses of TiO_2_ nanoparticles. (a) SEM MAG: 50kx, (b) SEM MAG: 200kx.

The SEM was employed to investigate the microstructural morphology of TiO_2_ nanoparticles, as illustrated in Fig 3. These SEM images were captured at magnifications of 50,000 and 200,000, respectively. The results revealed that the nanoparticles exhibited no apparent agglomeration and possessed good stability.

#### Preparation of oil samples with different water contents and temperatures

The prepared pure oil and nanofluid samples are placed in a constant temperature and humidity chamber, with a set temperature of 20°C. To obtain oil samples with different water content, the humidity of the constant temperature and humidity chamber is adjusted using a water absorption method to control the water content in the oil samples. To ensure the experiment closely resembles practical conditions, the water content in the oil samples is controlled at three levels: low water content of 26 ppm, medium water content of 61 ppm, and high water content of 85 ppm. Multiple measurements are taken for each water content level, and the average value is calculated to ensure an error within a range of ±1 ppm. To obtain oil samples at different temperatures, a programmable constant temperature and humidity box was used to control the temperature of oil samples with different water contents. A total of 6 temperature levels were tested in this experiment. Each temperature control time was not less than 18 hours, and the error of different temperatures was within ± 1.2°C.

### Experimental platform building

**Fig 4:**
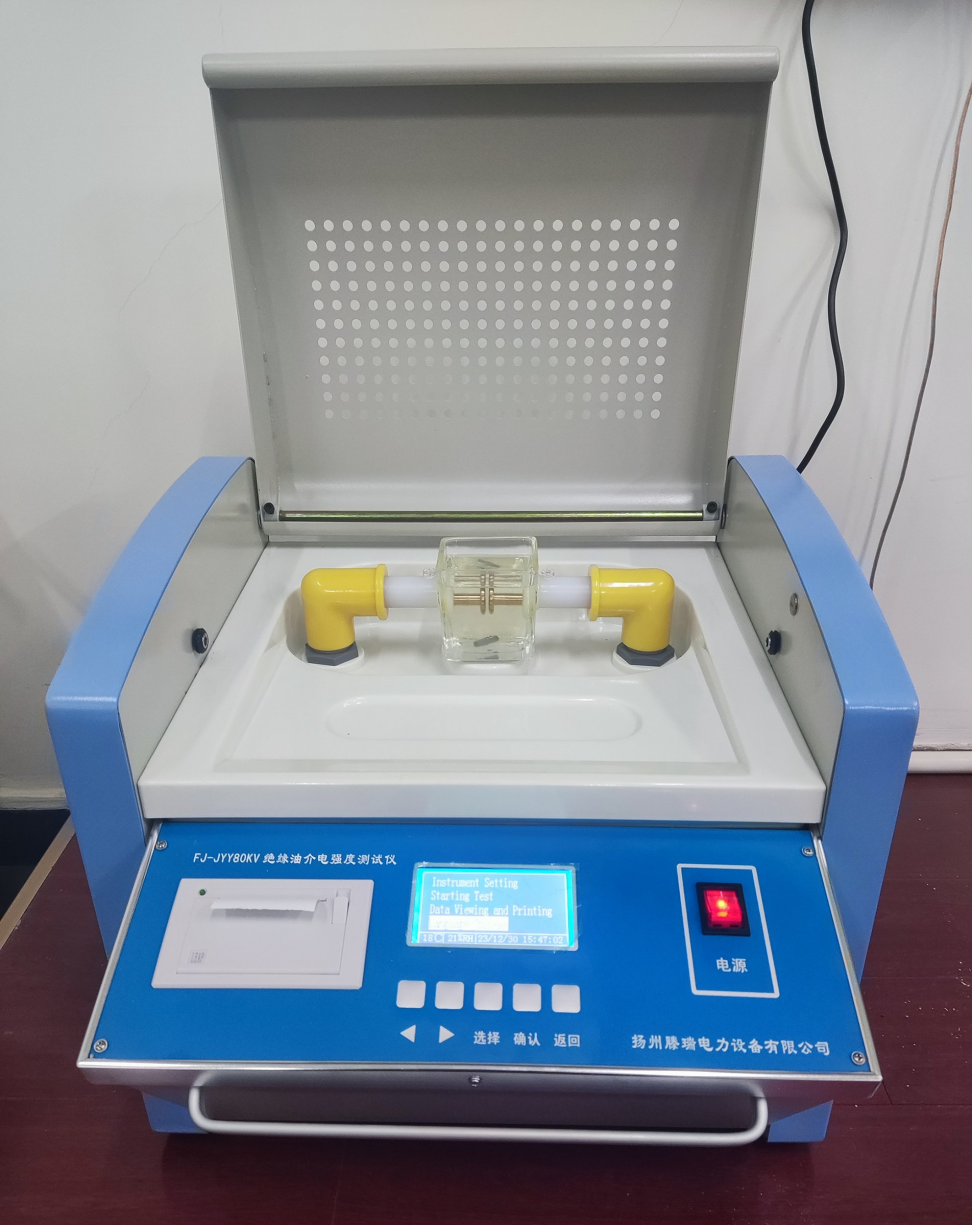
FJ-JYY80KV insulation oil pressure tester.

**Fig 5:**
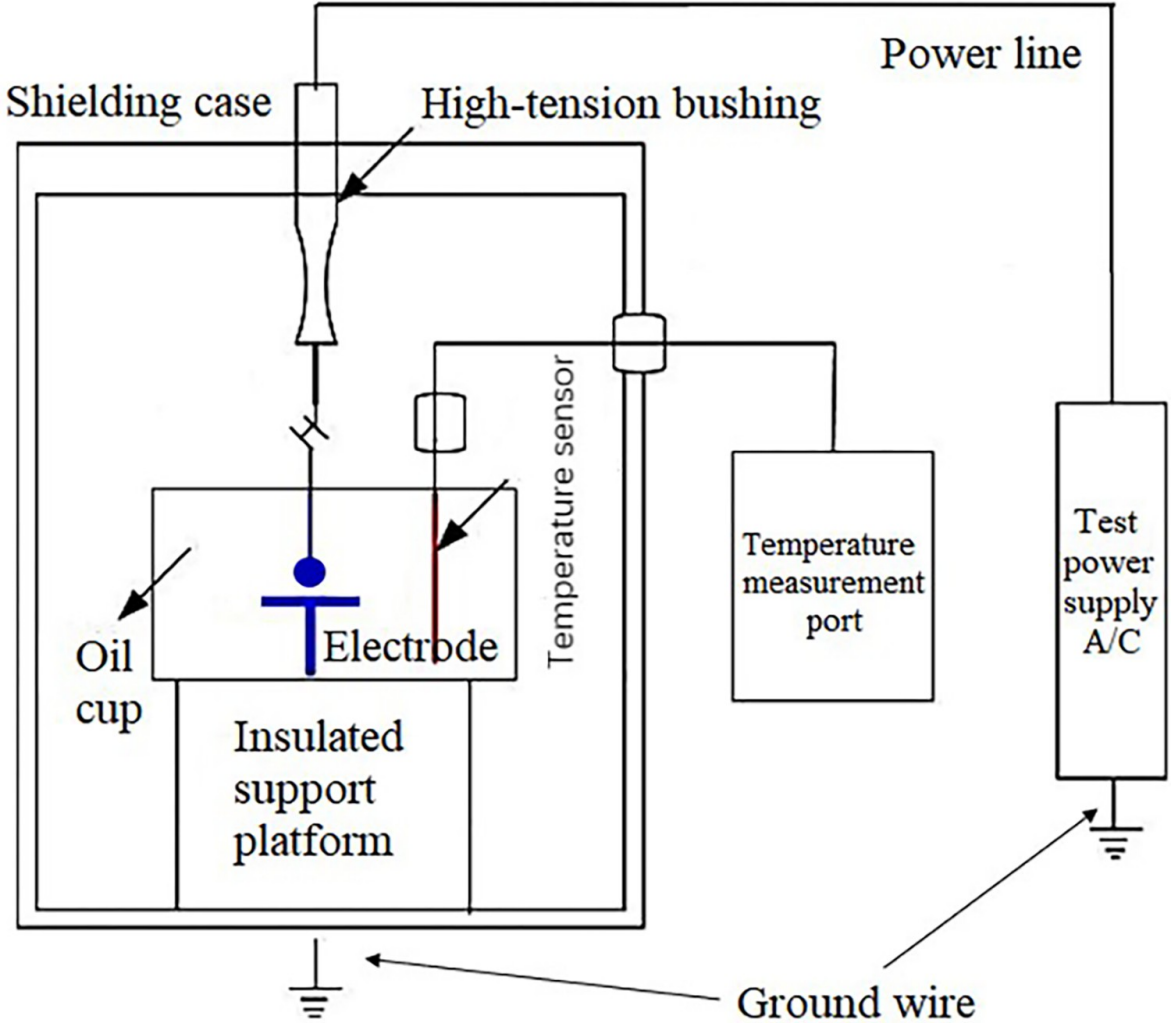
Insulation oil withstands voltage tester.

The transformer oil breakdown test platform is selected as the FJ-JYY80KV insulating oil withstand voltage tester shown in Fig 4, which can accurately withstand voltage testing for insulating liquids such as mineral oil, synthetic ester oil, and silicone oil. The test container module provides reliable data with good repeatability by locking the adjustment wheel for the precise electrode gap. Its internal principle is illustrated in Fig 5, with an external power supply of AC frequency, one electrode connected to a high-voltage bushing, and the other connected to an oil cup. The oil cup has a capacity of 400 mL, with a maximum breakdown voltage of 100 kV, and a protection action time of less than 10 ms. The oil cup is placed on an insulating platform, equipped with an internal temperature fiber sensor, and covered with a transparent acrylic electrostatic shielding cover to ensure the safety of the test personnel and facilitate the observation of the entire breakdown process. A cup cover is placed on the oil cup to seal it to some extent, preventing the oil surface from contacting air during the test and affecting the test results.

This tester can accurately withstand voltage testing for insulating oils such as mineral oil, synthetic esters, and silicone oil. The test container module provides reliable data with good repeatability by locking the adjustment wheel for the precise electrode gap.

The primary technical parameters of the instrument are as follows: Output voltage: 0 to 80 kV; Voltage rising rate: 0.5 to 5 kV/s; Number of voltage rising cycles: 1 to 9; Voltage distortion rate: <3%; Measurement accuracy: ±3%.

**Fig 6:**
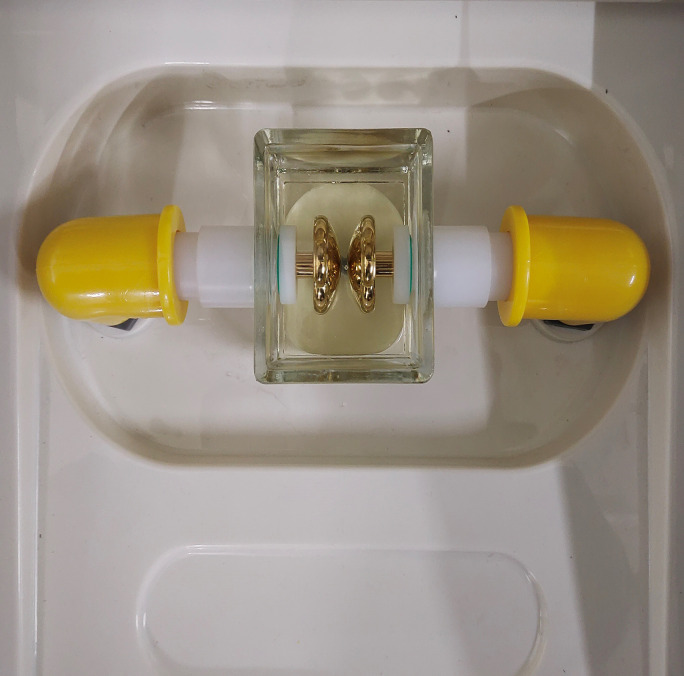
Test oil cup and electrode physical diagram.

The breakdown test was conducted strictly by the IEC-60296-2020 international standard. The physical images of the oil cup and electrodes used in the test are shown in Fig 6. For the breakdown test, a copper spherical cover electrode was selected to simulate a slightly non-uniform electric field. A spring was built into the electrode connecting rod within the oil cup to adjust the electrode spacing to 2.5 mm, and the spacing was fixed on the outside connecting rod with a screw. During the breakdown test, the test personnel should maintain a certain distance from the equipment, and the entire test process should be conducted indoors.

The transformer oil temperature control platform consists of the DW-50 low-temperature test chamber produced by Yuecha Company and the XMYA-500T electrically heated thermostatic chamber produced by Supo Company. The primary technical specifications of the low-temperature test chamber are as follows: temperature range of 10 to -50°C, resolution of 0.1°C, temperature fluctuation of ±2°C, and cooling rate of 1°C per minute. The primary technical specifications of the electrically heated thermostatic chamber are as follows: temperature range of 10 to 300°C, resolution of 1°C, and temperature control accuracy of ±1%.

### Experimental method and steps

1. Pour the test oil sample into the oil cup quickly, adjust the position of the magnetic stirring rod on the inner wall of the oil cup, and close the top cover of the oil cup and the electrostatic shield.
2. Select the test standard and set the voltage to a 61.8 Hz AC voltage. Gradually increase the voltage in steps of 2 kV/s until the oil gap breaks down.
3. Let it stand for 2 minutes. During the standing time, use the magnetic stirring rod to stir the oil sample to ensure the oil in the cup remains uniform. Perform another breakdown test. After 12 breakdowns, calculate the average value.

## Experimental results and analysis

The breakdown test data is analyzed using the Weibull distribution function, which provides a visualization image of the breakdown probability distribution of nanomodified transformer oil and pure oil, allowing for more accurate prediction of the breakdown voltage at 50% probability and lower probabilities [35, 36]. The experimental results of pure oil and nanofluid at different water content levels and temperatures are fitted with the Weibull distribution to observe the effect of nanoparticles on the breakdown voltage of transformer oil.

### Analysis of the influence of nanoparticles on breakdown voltage

**Fig 7:**
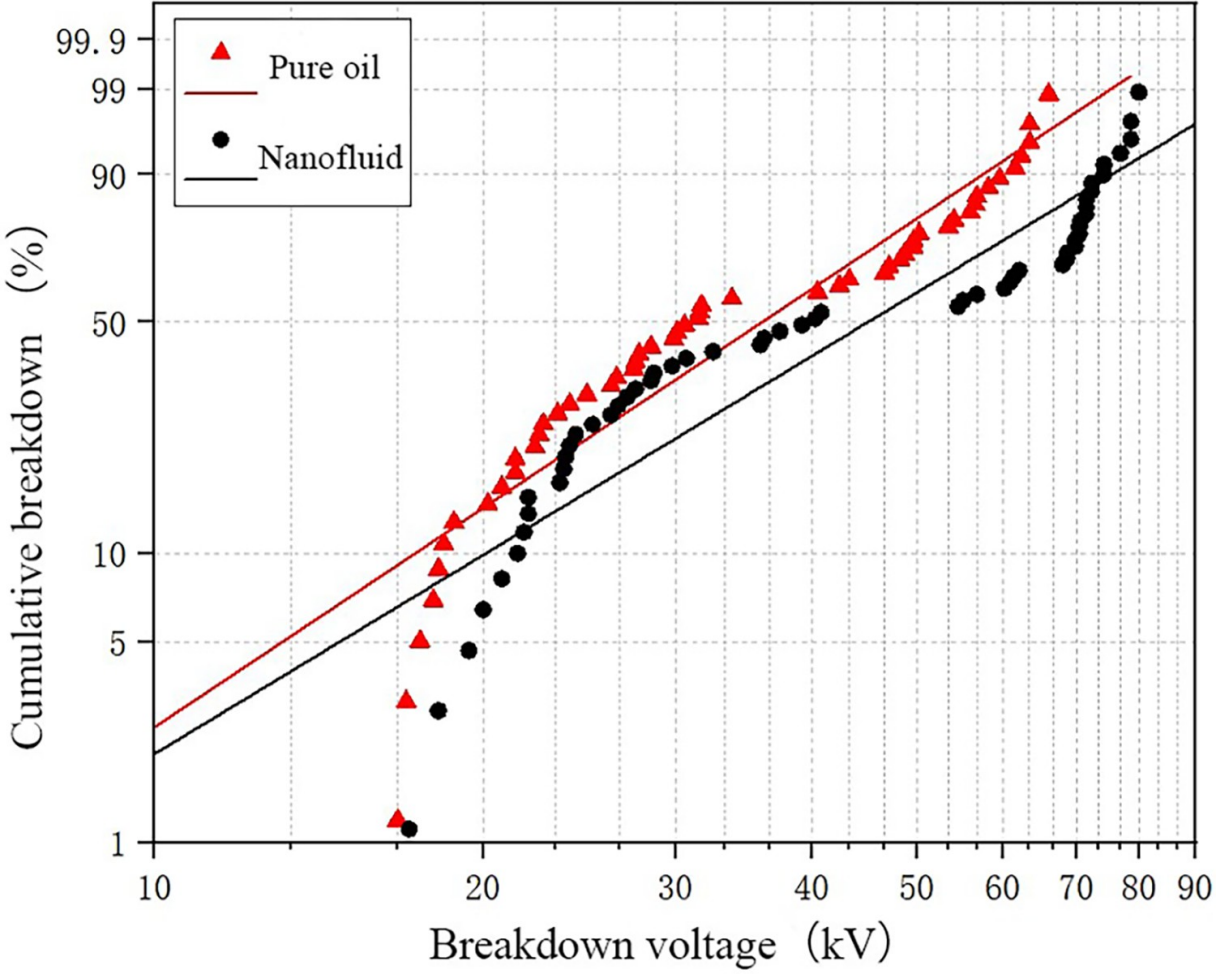
The fitting results of WEIBULL function under low water content.

The breakdown data for water content of 26 ppm is fitted and shown in Fig 7, which reveals that nanomodified transformer oil has a higher breakdown voltage at the same breakdown probability. In the part with higher breakdown voltage, the improvement of nanofluid is more obvious, while it is relatively smaller in the lower region. This indicates that the higher the breakdown voltage of pure oil, the more significant the improvement in breakdown voltage achieved by nanoparticles.

**Fig 8:**
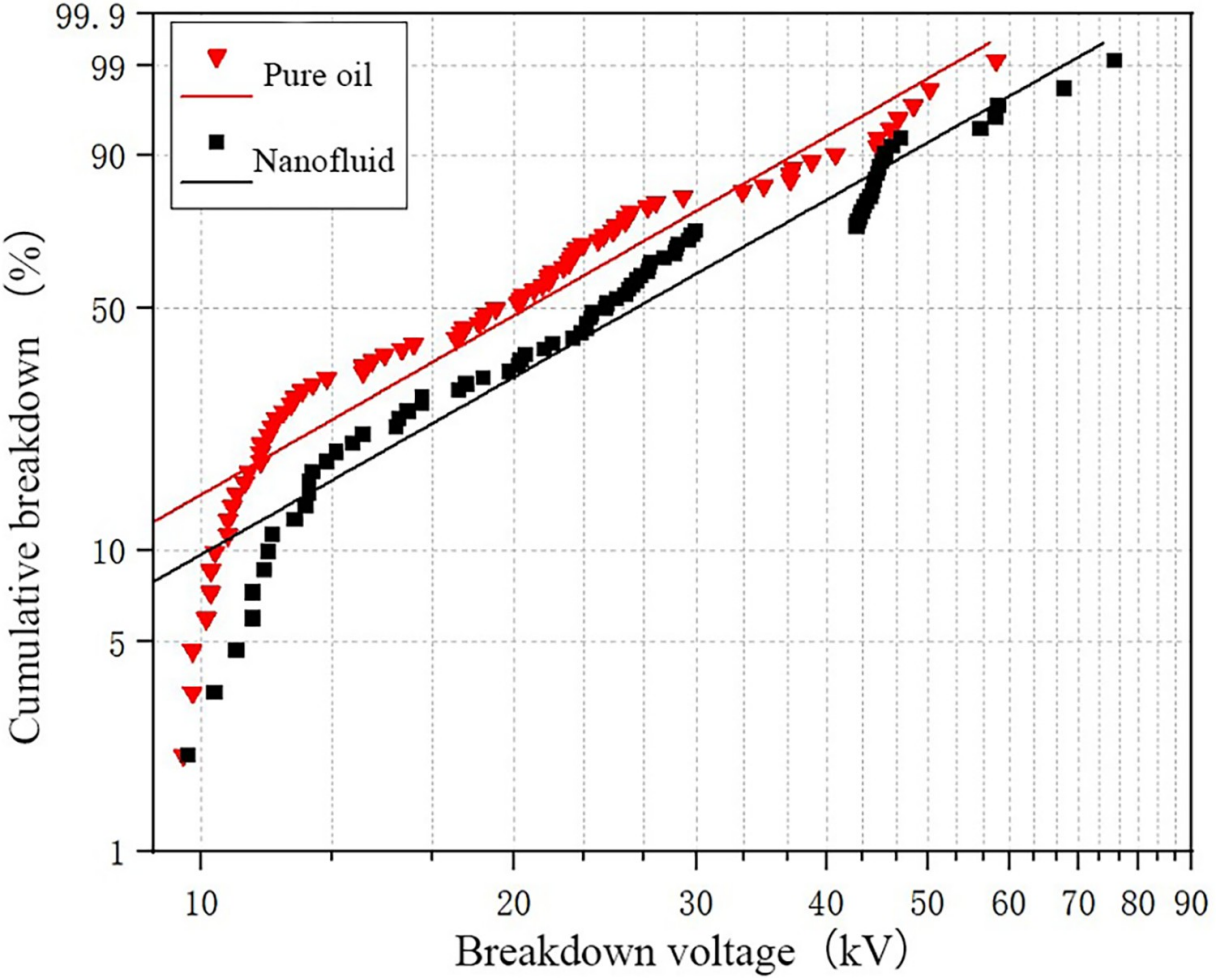
The fitting results of WEIBULL function under medium water content.

Fig 8 shows the fitting results of the Weibull function for a water content of 61 ppm. Compared to the fitting results in Fig 7, the overall curve shifts to the left, indicating that as the water content increases, the breakdown probability of the oil gap gradually increases, and the breakdown voltage decreases. From the Fig 8, it can also be observed that the gap between the fitting curves of the two oil samples decreases, indicating that the enhancement effect of nanoparticles on the breakdown voltage of transformer oil decreases as the water content in the oil increases.

**Fig 9:**
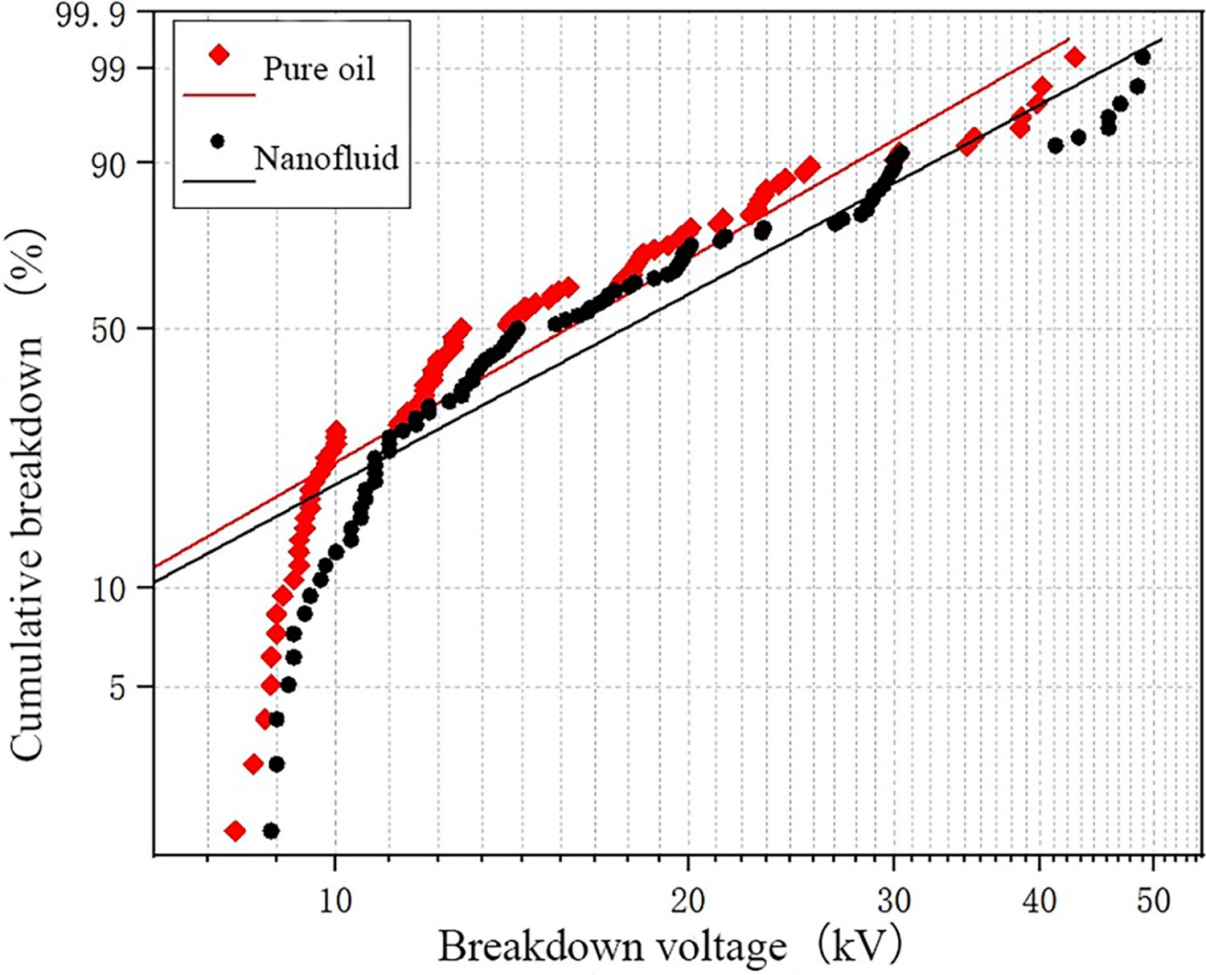
The fitting results of WEIBULL function under high water content.

Fig 9 presents the Weibull distribution fitting data for water content of 85 ppm. It can be seen that the breakdown voltage decreases significantly at this water content level, indicating a significant influence of water content on the breakdown voltage of transformer oil. When the breakdown probability is the same, the breakdown voltage of the nanofluid has a slight increase. At this water content level, the 5% probability breakdown voltage of nanofluid is 1.07 times that of pure oil, and the 50% probability breakdown voltage of nanofluid is 1.20 times that of pure oil.

Table 3 provides the statistical results of breakdown voltage at different breakdown probabilities. According to the table, the breakdown voltage at 5% probability for nanofluid is 1.11 times that of pure oil, while the breakdown voltage at 50% probability for nanofluid is 1.28 times that of pure oil. This indicates that the addition of nanoparticles within the experimental range can improve transformer oil’s breakdown voltage. Table 4 presents the parameters obtained from the Weibull distribution fitting curve.

**Table 3:** Statistical results of breakdown voltage under different breakdown probabilities.

Test oil samples	Water content	5% probability breakdown voltage / kV	10% probability breakdown voltage / kV	20% probability breakdown voltage / kV	30% probability breakdown voltage / kV	40% probability breakdown voltage / kV	50% probability breakdown voltage / kV
**Pure oil**	26ppm	17.5	18.4	21.4	24.9	27.8	31.5
**Nanofluid**		19.4	21.5	23.8	26.6	30.7	40.3
**Pure oil**	61ppm	9.8	10.3	11.4	12.5	16.0	19.2
**Nanofluid**		11.2	11.7	13.5	17.7	21.8	24.5
**Pure oil**	85ppm	8.5	9.2	9.6	11.5	12.1	12.8
**Nanofluid**		9.1	9.7	10.8	11.7	13.2	15.3

**Table 4:** Parameter fitting values in the WEIBULL function.

Test oil samples	Water content	Shape parameter	Scale parameters
**Pure oil**	26ppm	2.57	41.55
**Nanofluid**		2.33	52.74
**Pure oil**	61ppm	2.02	24.62
**Nanofluid**		1.98	31.40
**Pure oil**	85ppm	2.13	18.73
**Nanofluid**		1.99	21.21

Fitting the test results of both oils with the Weibull function allows for a visual representation of the enhancement effect of nanoparticles on the breakdown voltage of pure oil. As the water content increases, nanoparticles’ enhancement effect on transformer oil’s breakdown voltage gradually decreases. The addition of nanoparticles can improve the breakdown voltage of transformer oil to a certain extent, enhancing the operational reliability of transformer oil in practical engineering applications.

### Analysis of the influence of temperature on breakdown voltage

Temperature significantly impacts the performance of transformer oil, especially on the breakdown voltage. When the temperature is high, the breakdown voltage increases. The test data at different temperatures is fitted using the Weibull function to analyze the influence of temperature on the breakdown voltage.

**Fig 10:**
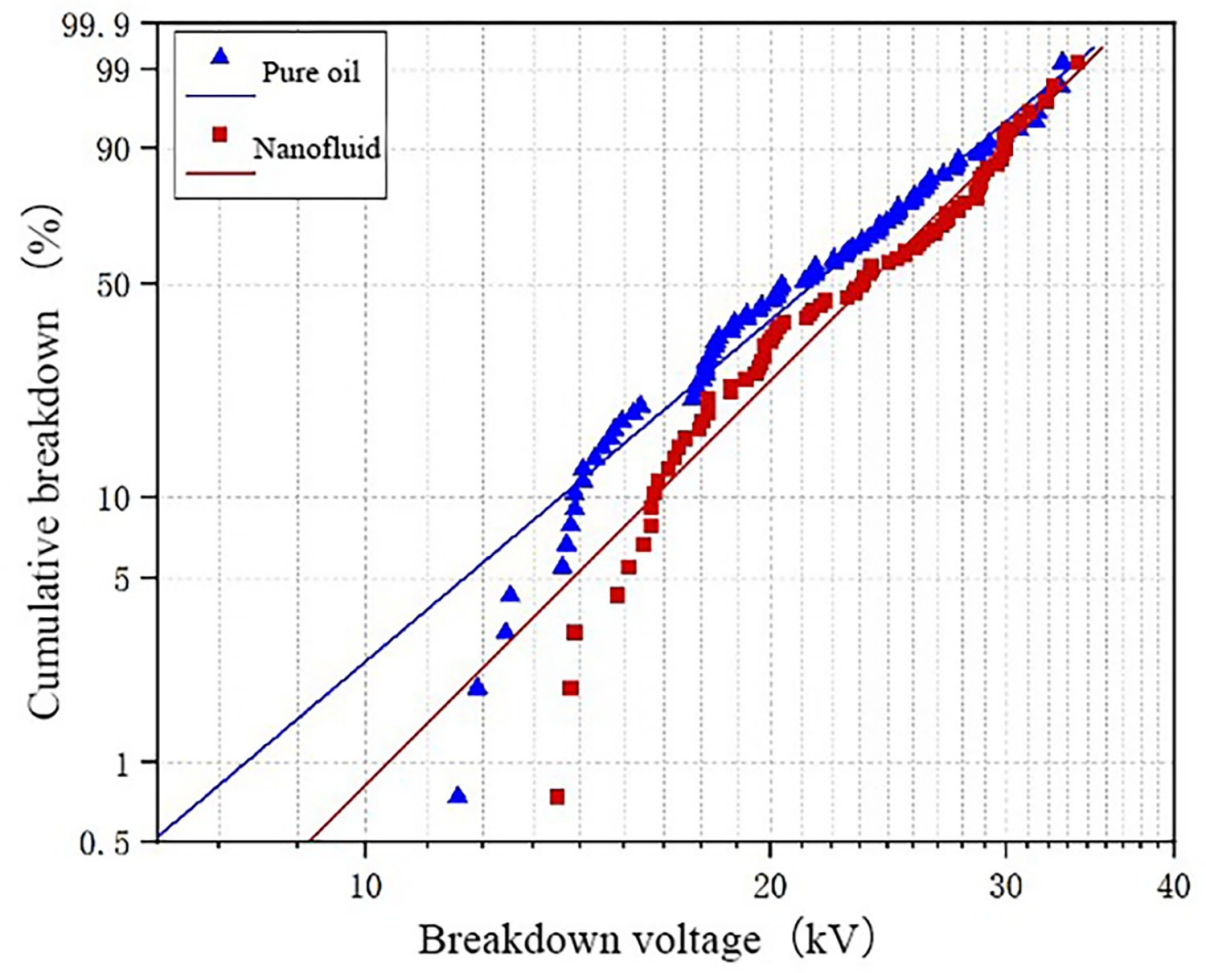
WEIBULL function fitting results of -30°C and -20°C test data.

The WEIBULL function fitting is performed on the test data at temperatures of − 30° C and − 20° C. The results are shown in Fig 10. It can be seen that the breakdown voltage of pure oil is low when the temperature is extremely low. On the whole, the slope of the fitting curve of nanofluid is higher than that of pure oil, which indicates that the breakdown voltage of nanofluid is higher than that of pure oil under the same breakdown probability, indicating that nano-materials have a certain improvement on the breakdown voltage of transformer oil at low temperature.

**Fig 11:**
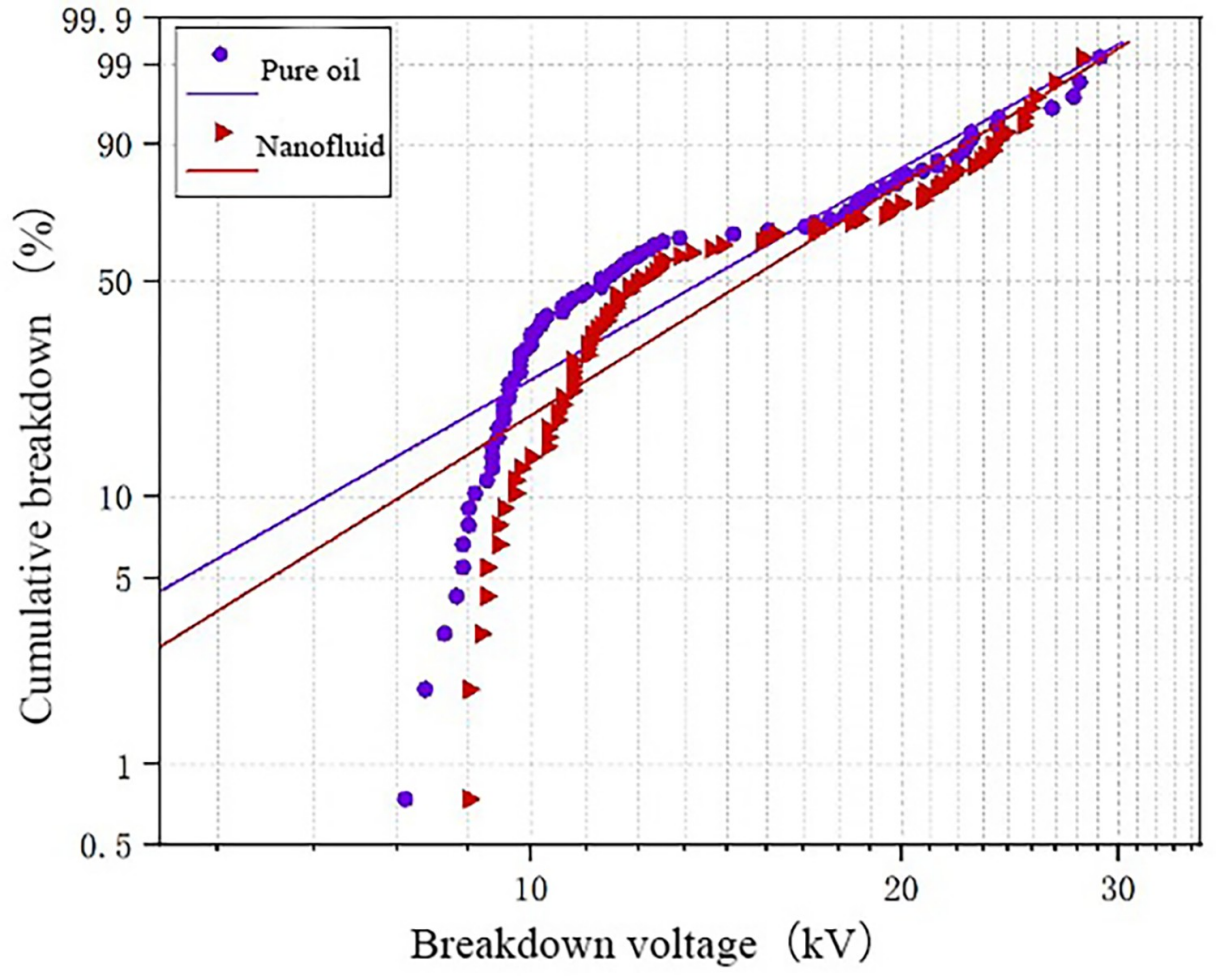
WEIBULL function fitting results of -10°C and 0°C test data.

Fig 11 presents the Weibull function fitting results for temperatures of -10°C and 0°C for both oil samples. From the Fig, it can be observed that within this temperature range, the enhancement effect of nanoparticles on the breakdown voltage of transformer oil is minimal. This is attributed to the form of water in the oil being suspended in this temperature range. This form of micro water significantly reduces the breakdown voltage of the oil gap. Consequently, the improvement in the breakdown voltage of transformer oil by nanoparticles is also minimal.

**Fig 12:**
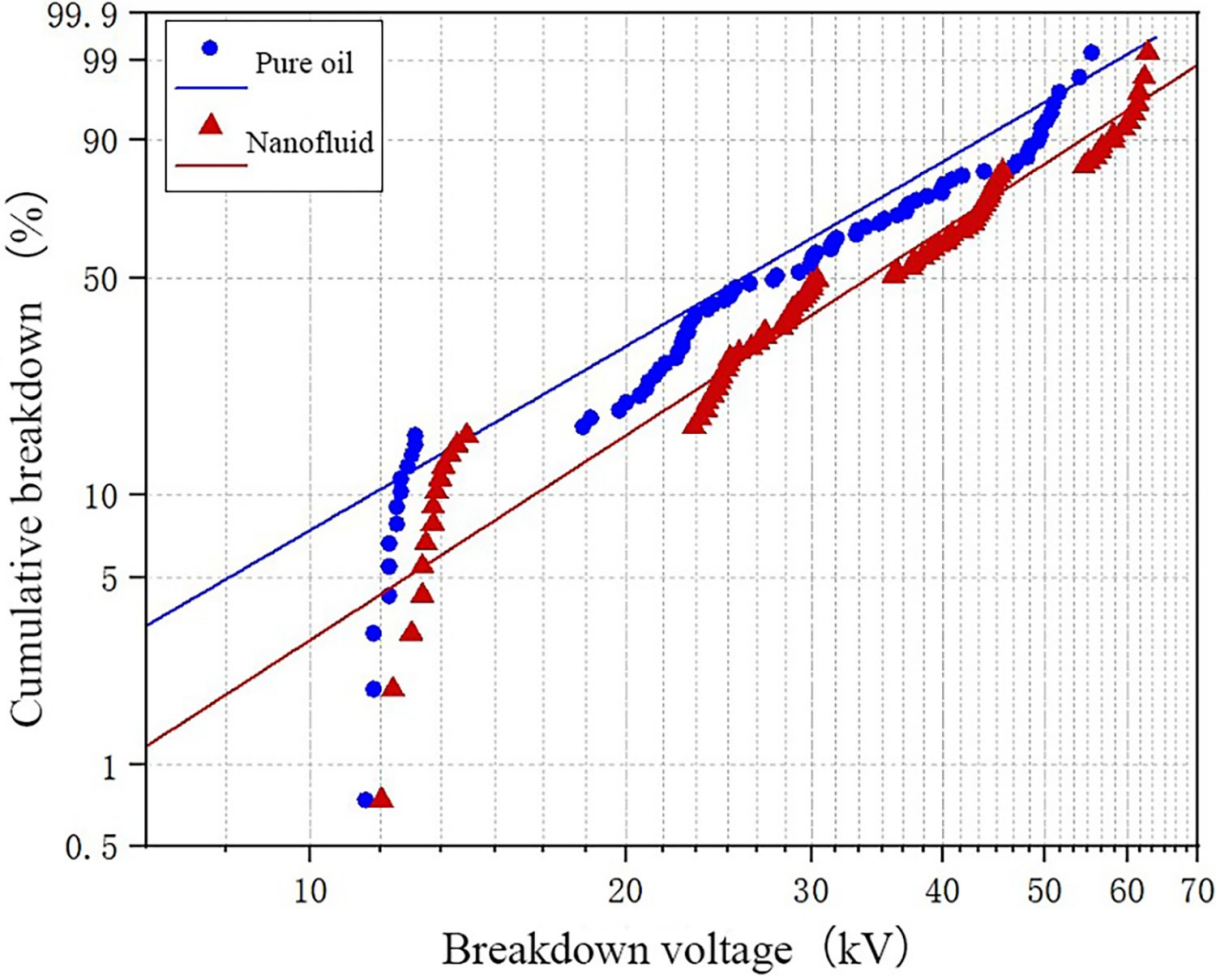
WEIBULL function fitting results of 10°C and 20°C test data.

The test data at 10°C and 20°C is fitted with the Weibull function, as shown in Fig 12. By observing the fitting curves in the Fig, it can be concluded that nanomodified transformer oil significantly increases breakdown voltage compared to pure oil. By comparing Figs 10 and 12, it can also be observed that the enhancement effect of nanoparticles on the breakdown voltage of transformer oil improves as the temperature increases.

Based on the above data analysis, it can be concluded that nanoparticles’ enhancement effect on transformer oil’s breakdown voltage varies with temperature. The effect is best at higher temperatures, followed by a lesser effect as the temperature decreases. The enhancement effect is least significant at temperatures between -10°C and 0°C. The fitting results obtained from the Weibull function align with the experimental results.

### Influence of nanoparticles on the resistivity of transformer oil

**Fig 13:**
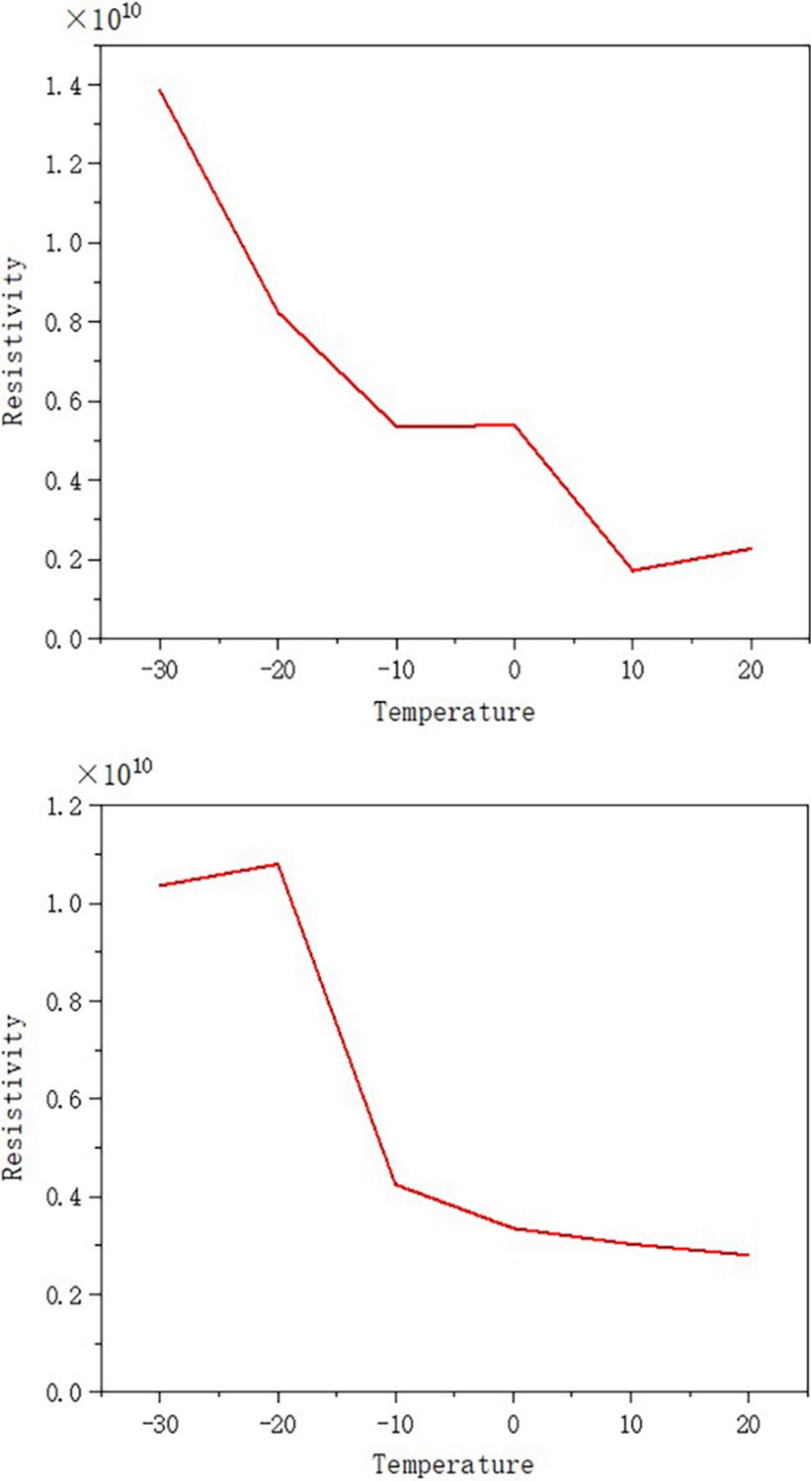
Curves of resistivity versus temperature for different oil samples. (a) Resistivity versus temperature curve for transformer oil, (b) Resistivity versus temperature curve for nanofluid.

The resistivity of nanofluid with a concentration of 0.01g/L and pure transformer oil was measured, and the curves showing the resistivity variation with temperature are presented in Fig 13.

Upon the addition of TiO_2_ nanoparticles into transformer oil, the nanofluid exhibited a lower electrical resistivity than the pure transformer oil due to the lower resistivity of the nanoparticles relative to the oil. As the temperature increased, both the resistivity of the pure transformer oil and the nanofluid decreased; at 20°C, the resistivity of the transformer oil was slightly lower than that of the nanofluid. This is attributed to the fact that transformer oil, as a dielectric, primarily conducts electricity through ionic conduction, and its conductivity increases with temperature, resulting in a decrease in resistivity as temperature rises. Initially, after the addition of nanoparticles, the resistivity of the transformer oil decreased significantly; however, with increasing temperature, the influence of the nanoparticles on the resistivity diminished progressively. This is in agreement with the experimental results, which reveal that as the temperature increases, the nanoparticles exhibit a more significant enhancement in the breakdown voltage of transformer oil.

### Analysis of the mechanism of nanoparticles enhancing oil breakdown voltage

Under the action of voltage, some of the ionized electrons at the negative electrode and the injected electrons from the cathode are adsorbed by transformer oil molecules to form negative ions. Another portion combines with positive ions, while the remaining electrons are continuously accelerated by the electric field and collide with molecules in the oil, generating an electron avalanche. The head of the electron avalanche primarily consists of negatively charged electrons, which strengthen the electric field between the head of the electron avalanche and the opposite positive electrode. The tail of the electron avalanche mainly consists of positive ions, which weaken the electric field inside the avalanche. Due to the recombination within the electron avalanche, photons are released, causing photoelectron ionization and generating a secondary electron avalanche. The secondary electron avalanche continuously merges with the initial electron avalanche, forming a streamer, which ultimately leads to the breakdown of the transformer oil.

Guo et al. found through experimental studies that nanoparticles enhance the onset and cutoff voltages of positive streamers while reducing the onset and cutoff voltages of negative streamers, thereby diminishing the polarity effect in transformer oil. The polarity effect is primarily caused by the differential mobility of electrons and ions within the transformer oil: electrons possess high mobility, whereas ion mobility is relatively low. Consequently, the positive and negative charges at the head of the streamer are separated due to their differing mobilities, which in turn influences the local electric field distribution and results in the polarity effect. Based on the significant role of nanoparticles in mitigating the polarity effect, it is inferred that nanoparticles slow down the migration rate of electrons in insulating oil [37, 38].

Considering that the migration rate of electrons in a streamer is much greater than that of positive ions when there are no nanoparticles present in the transformer oil, the centers of positive and negative charges will separate rapidly, forming a space charge region at the head of the streamer. This enhances the electric field strength between the streamer head and the opposite electrode, promoting faster streamer development.

When nanoparticles are present in transformer oil, as per the polarization capture theory, under the influence of an electric field, the nanoparticles become polarized, and the positive charges induced on their surface capture high-speed electrons. The capture process ceases when the entire region of positive charges is filled with electrons. Following the capture of electrons by the nanoparticles, slow-moving negative charge particles are formed. Electrons that are captured by shallow traps are more likely to migrate between adjacent traps, facilitating their escape from the ionization zone. Upon escaping, these electrons may be neutralized or form negative ions, thereby weakening the recombination ionization process caused by electrons in the ionization zone and inhibiting the development of the electron avalanche [39]. After capturing electrons, nanoparticles serve to decelerate the migration of positive and negative charge centers in the streamer, reducing the distortion of the space charge. Additionally, due to their larger particle size and slower movement compared to electrons, negatively charged nanoparticles have a lower probability of collisional ionization in the oil. Furthermore, they enhance the recombination of positive and negative charges. By decreasing the migration velocity and electric field strength at the head of the streamer, nanoparticles suppress the development of the streamer and thereby increase the breakdown voltage.

Thus, although the addition of nanoparticles results in the resistivity of nanofluids being lower than that of transformer oil, this is because the incorporation of nanoparticles introduces a large number of shallow traps, which facilitate the escape of electrons from the ionization region, thereby weakening the collisionally-induced ionization between the free electrons and the oil molecules. Additionally, the polarization of the nanoparticles captures electrons, reducing the number of electrons in the ionization region and further weakening the collisionally induced ionization. Both of these effects mitigate the collisionally induced ionization, suppress the formation and development of the electron avalanche, and enhance the breakdown voltage of the dielectric medium [25].

## Simulation

In this study, the COMSOL 5.6 finite element simulation software was employed to construct an electrostatic field model, modeling the electrodes and oil gap sections. The movement of charged particles and the influence of adding nanoscale particles were investigated. Based on the simulation results combined with experimental data, the impact of nanoparticles on the breakdown voltage of the oil gap was analyzed.

### Two-dimensional model of the electrostatic field

**Fig 14:**
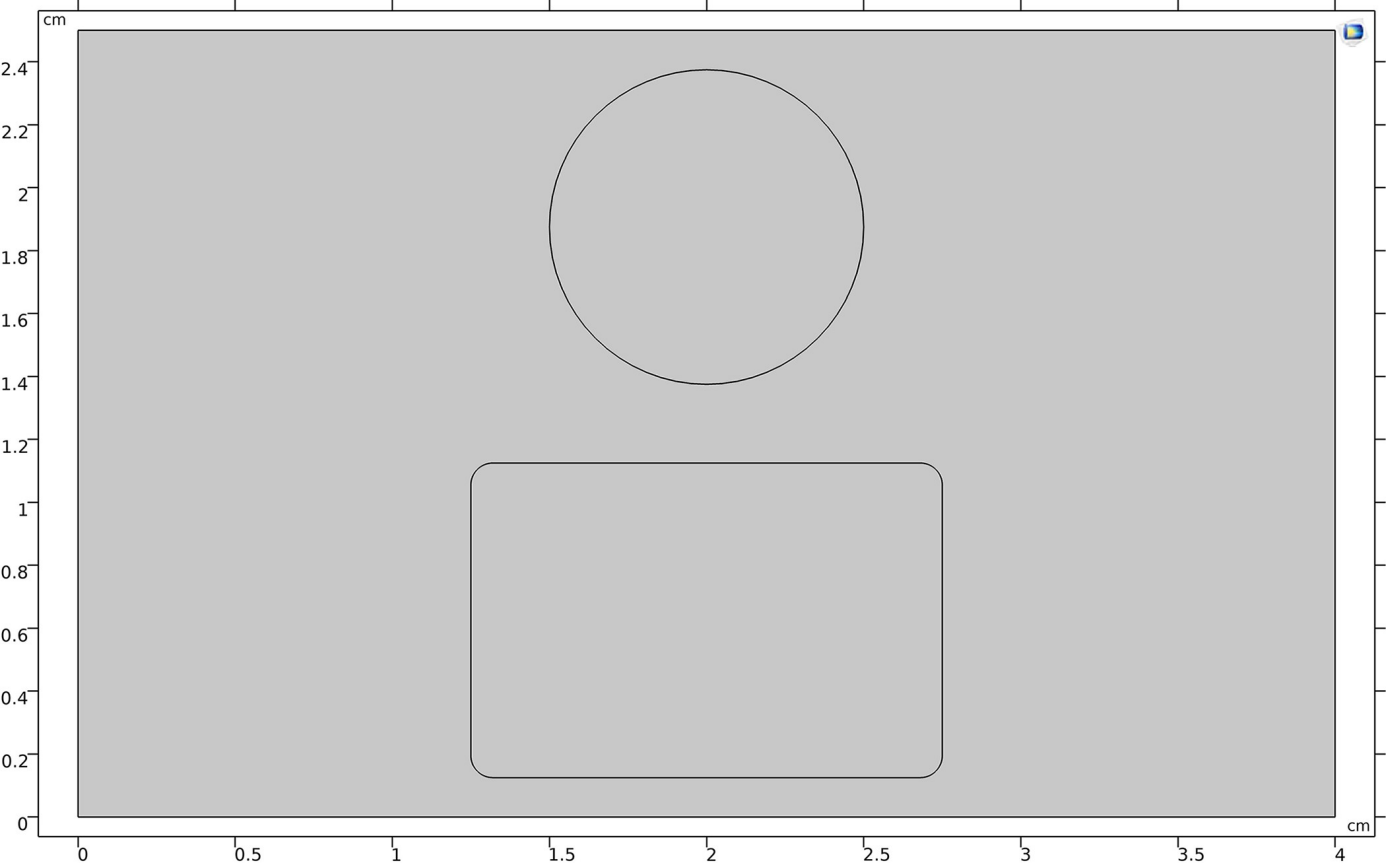
Two-dimensional model diagram of sphere plate electrode.

The simulation utilized an electrode structure combining spherical and flat electrodes to observe the trajectory of nanoparticles around different-shaped electrodes. A two-dimensional model of sphere plate electrode is shown in Fig 14, where the diameter of the spherical electrode is 1cm, the diameter of the flat electrode is 1.5cm, the electrode gap is 2.5mm, the electrode material is copper with a conductivity of 5.998×10^7^ S/m, a relative permittivity of 1, a reference resistivity of 1.72×10^−8^, and the insulating medium is transformer oil, with parameters set according to the properties of #45 transformer oil.

### Physical field control equations

#### Electrostatic field control equations

The transient equations of the electrostatic field are as follows [40]:

$$\nabla ∙D=\rho_{v}$$ (1)

E=−∇V (2)

The control equations of charge conservation are as follows [40]:

$$\nabla \cdot (\varepsilon_{0}\varepsilon_{r}E)=\rho_{V}$$ (3)

$$D=\varepsilon_{0}\varepsilon_{r}E$$ (4)

where *D* represents the displacement in the direction of the electric field; *ρ*_*v*_ represents the spatial charge density; *ε*_0_ represents the relative permittivity in a vacuum; *ε*_*r*_ represents the relative permittivity of the material; *E* represents the electric field strength, *V* represents the voltage.

#### Particle tracking control equations for fluid flow

The equations describing the forces exerted on particles and their motion velocities are as follows [41]:

$$\frac{d\;(m_{p}v)}{dt}=F_{t}$$ (5)

$$v=v_{c}-2(n∙v_{c})n$$ (6)

where *m*_*p*_ represents the particle mass; *v* represents the particle velocity; *v*_*c*_ denotes the velocity upon impact with the wall; *n* represents the unit vector in the normal direction of the interface between the two media.

The magnitude of the electric field force on the nanoparticles is, which can be mathematically expressed as follows:

$$F_{e}=eZE$$ (7)

The value of *e* is 1.602×10^−19^; *Z* is the number of charges; *E* represents the electric field strength.

The equation describing the dielectrophoretic force acting on the nanoparticles is as follows [42]:

$$F_{dep}=2\pi r_{p}^{3}\varepsilon_{0}\varepsilon_{r}Re[K(\omega )]\nabla |E{|}^{2}$$ (8)

$$K(\omega )=\frac{\varepsilon_{e}^{*}-\varepsilon_{i}^{*}}{\varepsilon_{e}^{*}+2\varepsilon_{i}^{*}}$$ (9)

where *r* represents the radius of the nanocrystal; *ε*_*i*_ represents the dielectric constant of insulating oil; *ε*_*e*_ represents the dielectric constant of nanoparticles; ε* = *ε*−*iσ*/*ω* represents electric permittivity; *σ* represents the electric conductivity; *ω* represents the angular frequency of the applied electric field; *m*_*p*_ represents the particle mass.

The equation describing the magnitude of the drag force on the nanoparticles during motion is as follows [43]:

$$F_{D}=\frac{1}{\tau_{p}}m_{p}\;(u-v)$$ (10)

$$\tau_{p}=\frac{\rho_{p}d_{p}^{2}}{18\mu }$$ (11)

where *u* represents the liquid velocity; *ρ*_*p*_ represents the fluid density; *d*_*p*_ represents the particle diameter; *μ* represents the liquid dynamic viscosity; *v* represents the velocity of particle motion.

The interaction force between particles describes the interaction between different charged particles and between charged particles and nanoparticles. The description equation is as follows [24]:

$$F=G{m_{p}}^{2}\sum_{j=1}^{N}\frac{r-r_{j}}{{\left|r-r_{j}\right|}^{3}}$$ (12)

where *r*_*j*_ represents the position vector of the jth particle; *F* represents the interaction force between any particle and the jth particle; *G* is the gravitational constant, and m is the mass.

For the release boundary conditions of particles, to simulate the arbitrary distribution of charged particles in the oil, the random release of particles is selected here, which is convenient to simulate the most realistic initial existence state of particles in the oil.

## Simulation results and analysis

**Fig 15:**
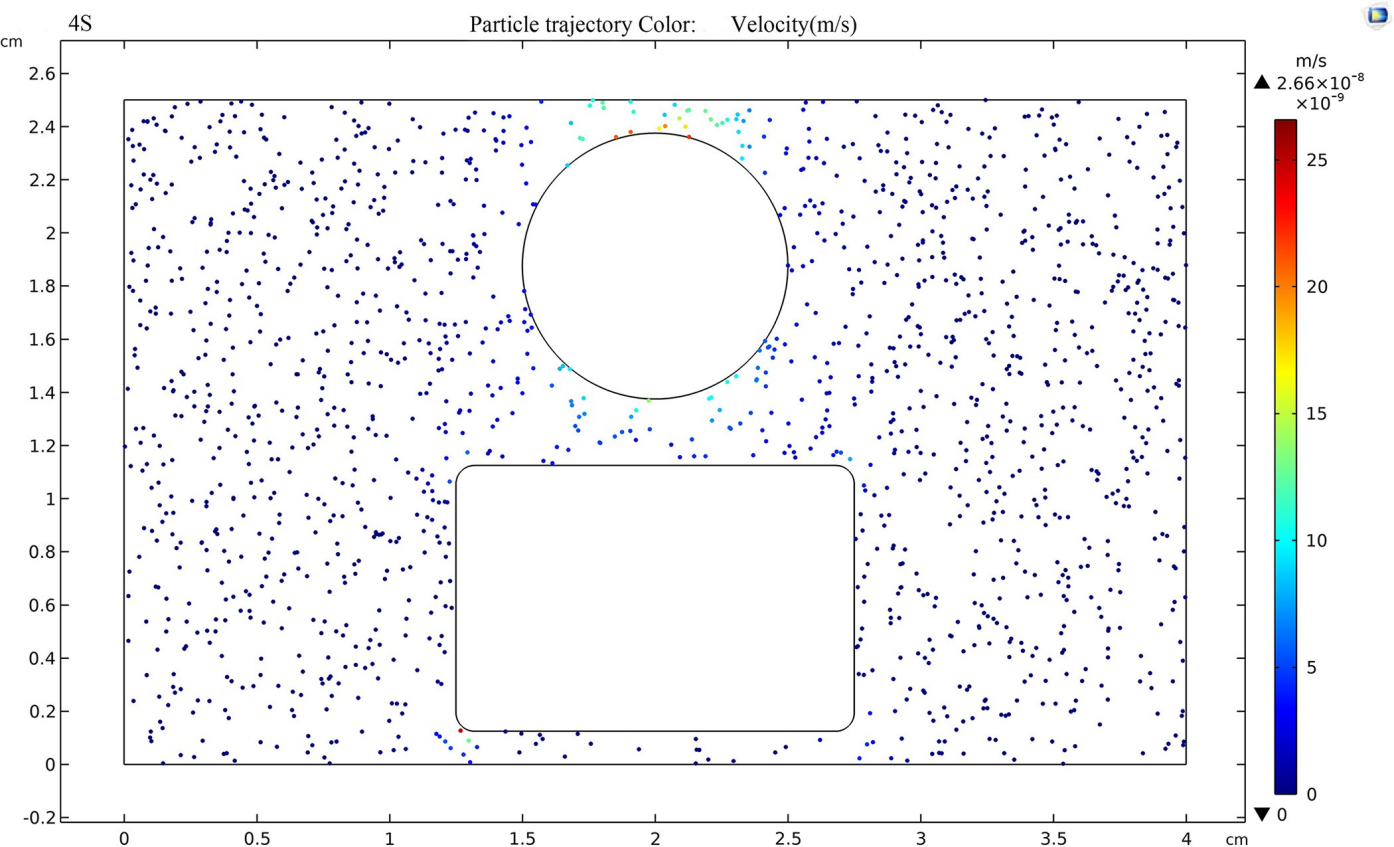
Trajectory of nanoparticles.

800 TiO_2_ nanoparticles with a diameter of 20nm were randomly released into the insulating oil under an applied DC voltage of 80kV. The trajectory of TiO_2_ nanoparticles under the electric field is shown in Fig 15, indicating that the movement of nanoparticles is extremely slow, with short distances that can be neglected. This is due to the smaller particle size of the nanoparticles and the smaller dielectrophoretic force on polarization, which is not sufficient to cause significant motion.

In transformer oil, the presence of nanoparticles causes a deviation in the electric field distribution around the nanoparticles. Under the electric field, TiO_2_ nanoparticles produce polarized charges on their surfaces, creating charge traps in the surrounding area. These traps capture nearby electrons, causing the nanoparticles to carry a charge. Thus, under the influence of the electric field, nanoparticles acquire a certain velocity, generating different trajectories. M. Zahn and others modeled the electrodynamics of nanoparticles in an electric field and proposed a conduction capture model based on the formation of potential traps after the polarization of nanoparticles by electrons, explaining the formation of nanoparticle traps and electron capture behavior. Studies indicate that the relative magnitude of the electron redistribution relaxation time in nanoparticle materials to the streamer development time is crucial for the development of streamer injection. The dielectric constants and conductivities of nanoparticles and transformer oil were introduced into Eq (13) to calculate the relaxation time of nanoparticles under the action of the electric field [44].

$$\tau =\frac{2\varepsilon_{i}+\varepsilon_{e}}{2\sigma_{i}+\sigma_{e}}$$ (13)

where *σ*_*i*_ represents the conductivity of insulating oil; *σ*_*e*_ represents the Conductivity of nanoparticles.

The relaxation time of TiO_2_ nanoparticles is 1.05 × 10^−3^ s. The streamer development time of TiO_2_ nano-modified transformer oil is 7 × 10^−7^ s, which is much smaller than the relaxation time. Therefore, it is considered that the charge trap of TiO_2_ nanoparticles is mainly formed by polarized charges. When the positive charge after polarization captures the electron, due to the short time, the electron still stays in the captured position, so on the surface of TiO_2_ nanoparticles, the electron presents a non-uniform distribution. Assuming that the polarized positive charges on the surface of TiO_2_ nanoparticles are completely neutralized by electrons, the captured electron density is [38]:

$$\sigma_{se}=3\varepsilon_{0}\frac{\varepsilon_{i}-\varepsilon_{e}}{2\varepsilon_{e}+\varepsilon_{i}}E_{0}cos\theta \;-\frac{\pi }{2}<\theta <\frac{\pi }{2}$$ (14)

At this time, the total charge absorbed by the nanoparticles is obtained from Eq (15).


$$Q_{s}(t<<\tau )=-\int_{-\frac{\pi }{2}}^{\frac{\pi }{2}}\begin{array}{c} \int_{0}^{\frac{\pi }{2}}2*3\varepsilon_{0}\frac{\varepsilon_{i}-\varepsilon_{e}}{2\varepsilon_{e}+\varepsilon_{i}}ER^{2}\;cos\theta sin\theta d\theta d\varphi \end{array}=-3\pi \varepsilon_{0}\frac{\varepsilon_{i}-\varepsilon_{e}}{2\varepsilon_{e}+\varepsilon_{i}}ER^{2}$$ (15)

**Fig 16:**
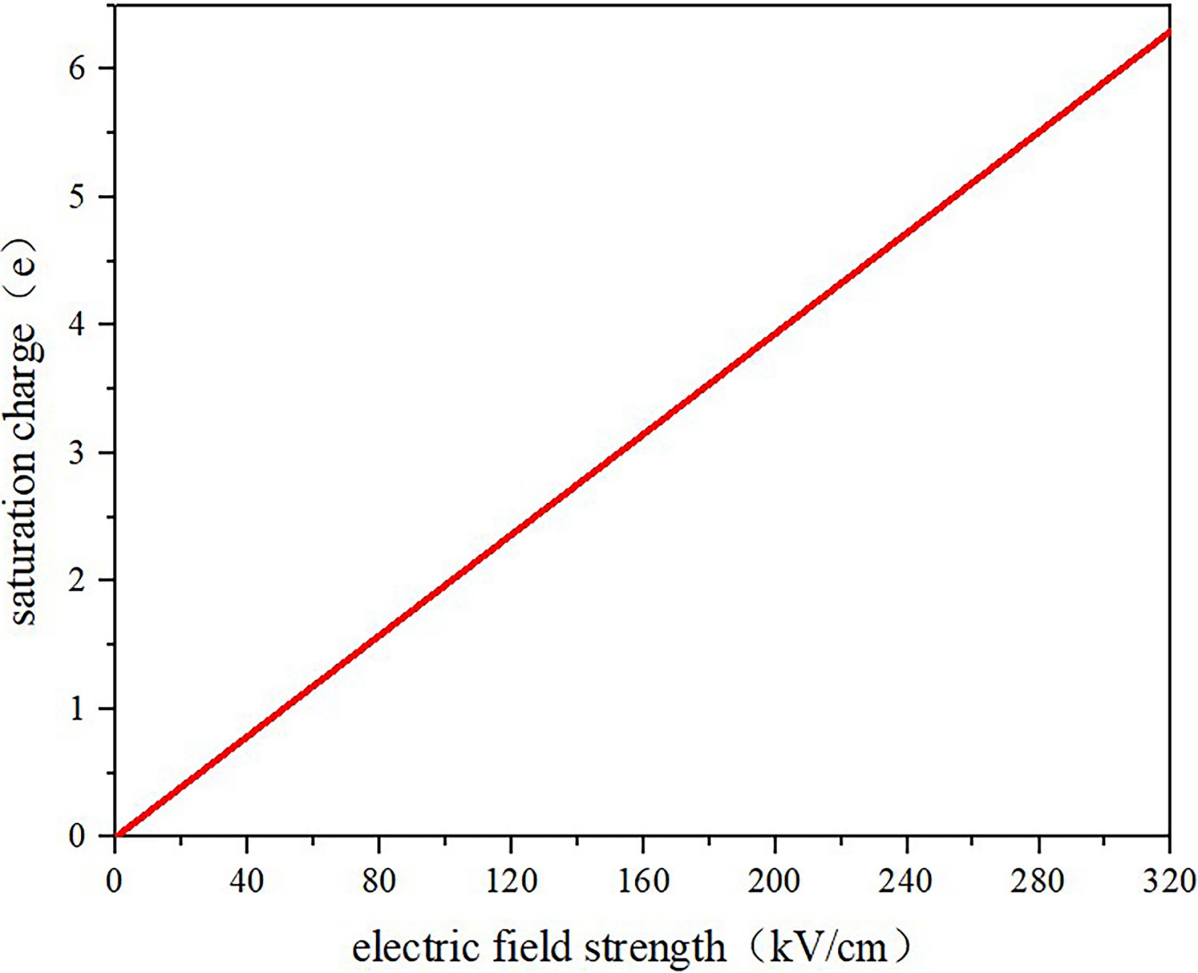
The saturated charge of TiO_2_ nanoparticles.

According to Eq (15), the saturated charge quantity of TiO_2_ nanoparticles under different electric field strengths is calculated, and the variation is plotted. From Fig 16, it can be observed that the saturated charge quantity of TiO_2_ nanoparticles linearly increases with the increase of the electric field strength. Moreover, according to Eq (15), under the premise of unchanged electric field strength, the larger the particle size of nanoparticles, the greater the saturated charge quantity, indicating that nanoparticles capture more electrons.

Based on the derivation results of Eq (14), different electric field strengths and the number of charges adsorbed by nanoparticles under different field strengths were input into COMSOL for simulation and the results are basically in line with the actual situation.

**Fig 17:**
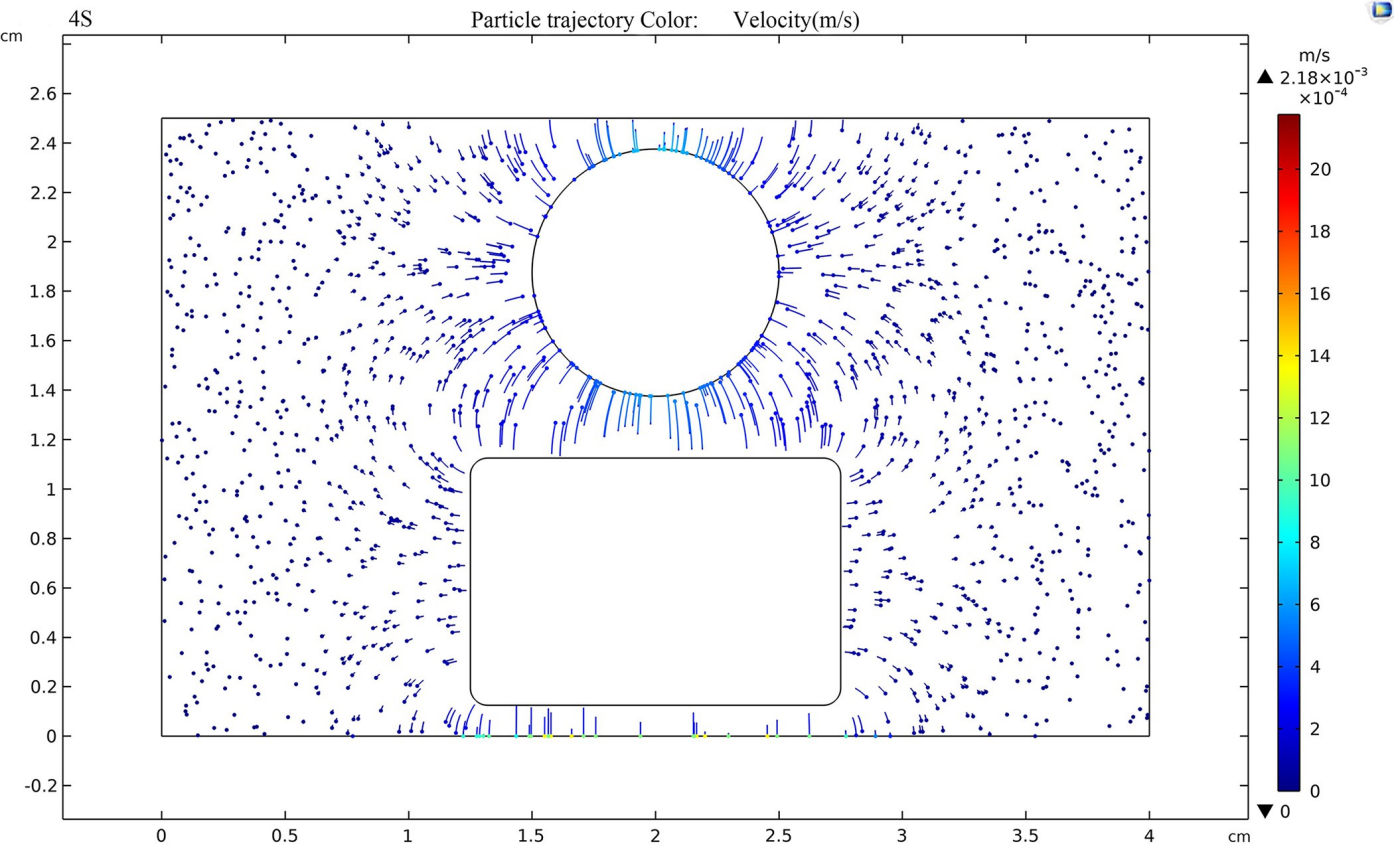
Trajectory of nanoparticles at an applied voltage of 20 kV.

Fig 17 shows the trajectory of TiO_2_ nanoparticles in the oil gap when an external voltage of 20kV is applied and the electric field strength between the sphere plate electrode is 80kV/cm. Tracking the TiO_2_ particles in nanofluid reveals that TiO_2_ particles, after adsorbing electrons, exhibit a negative charge and in the oil gap, there is a trend of moving from the negative electrode (plate pole) to the positive electrode (spherical pole), the fastest moving speed of nanoparticles near the electrode is 2.18×10^-7^m/s, this is because the electric field strength is highest near the electrodes, leading to the maximum force exerted on the nanoparticles in that vicinity.

**Fig 18:**
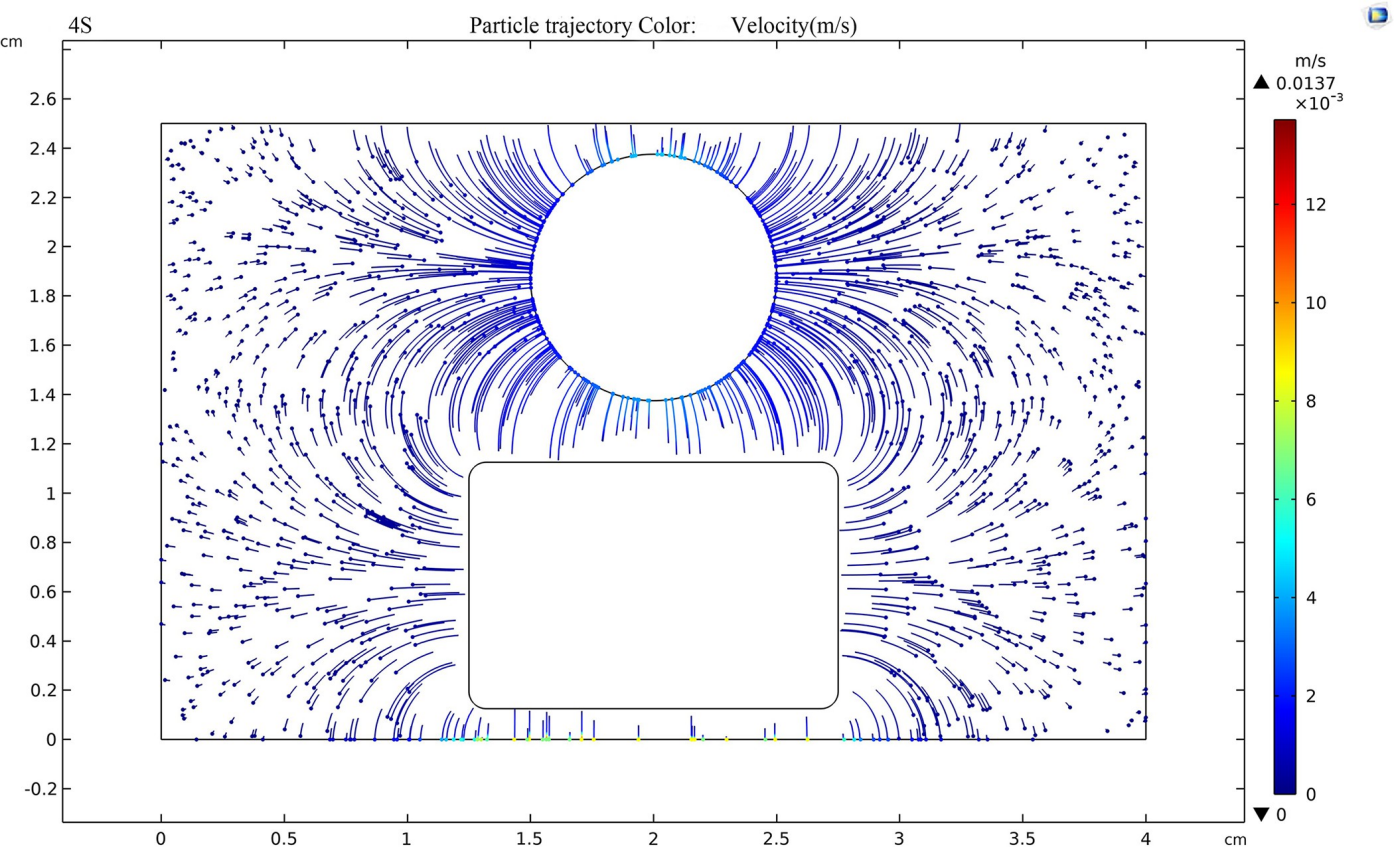
Trajectory of nanoparticles at an applied voltage of 50 kV.

As shown in Fig 18, when the external voltage is 50kV and the field strength in the electrode is 200kV/cm, the fastest movement speed of TiO_2_ nanoparticles in the oil gap is 1.37×10^-5^m/s. Compared with Fig 17, the speed increases by two orders of magnitude, and the movement trend of charged nanoparticles toward the positive electrode (sphere) becomes more apparent.

**Fig 19:**
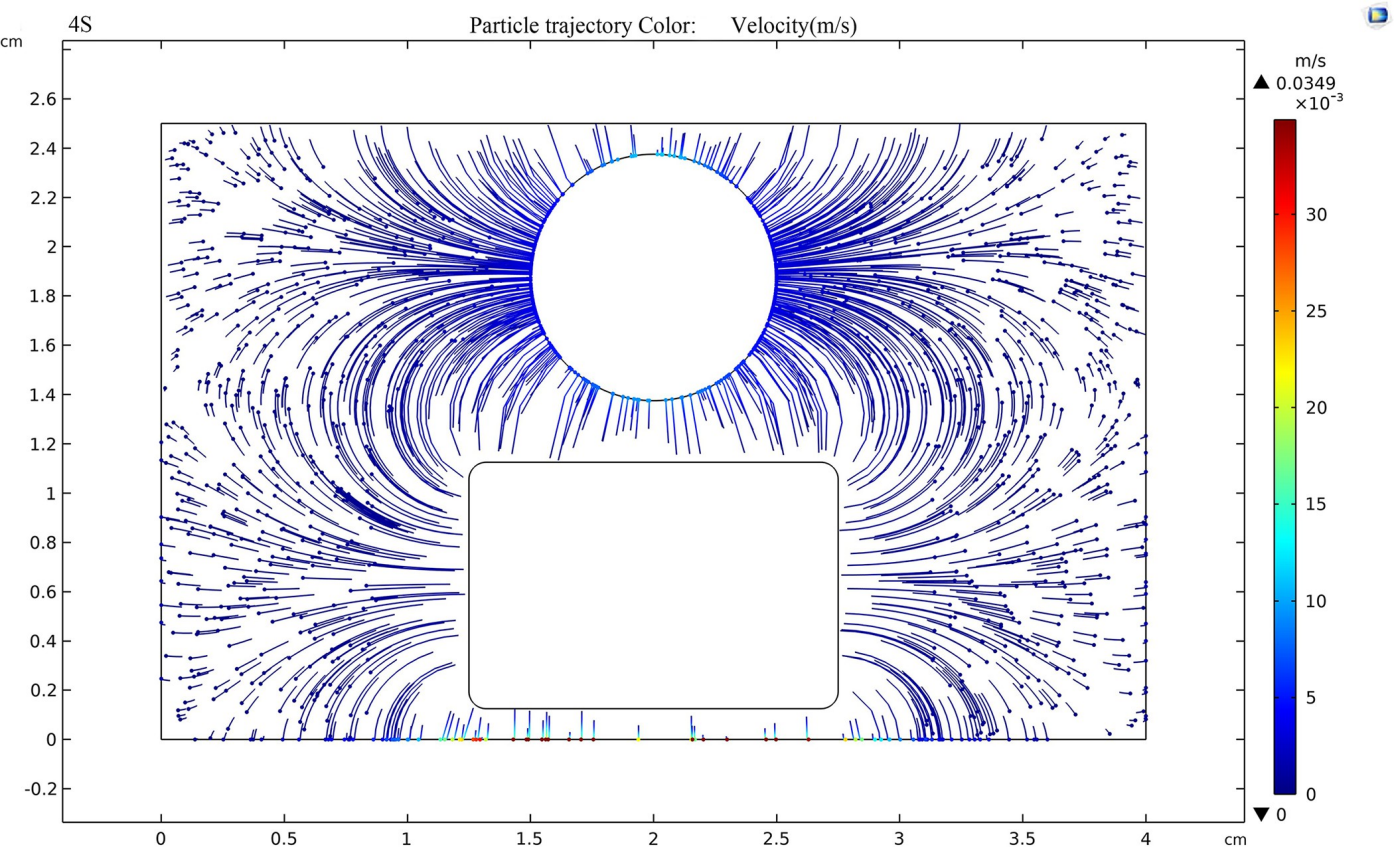
Trajectory of nanoparticles at an applied voltage of 80 kV.

According to Fig 19, when the external voltage is 80kV and the electric field strength between the sphere plate electrode is 320kV/cm, the movement trend of nanoparticles under the action of the electric field is very obvious. After stabilization, the speed of nanoparticles can reach 3.49×10^-5^m/s, which is 2.5 times higher than that at 50kV, the force of positive and negative electrodes on charged particles is maximized.

**Fig 20:**
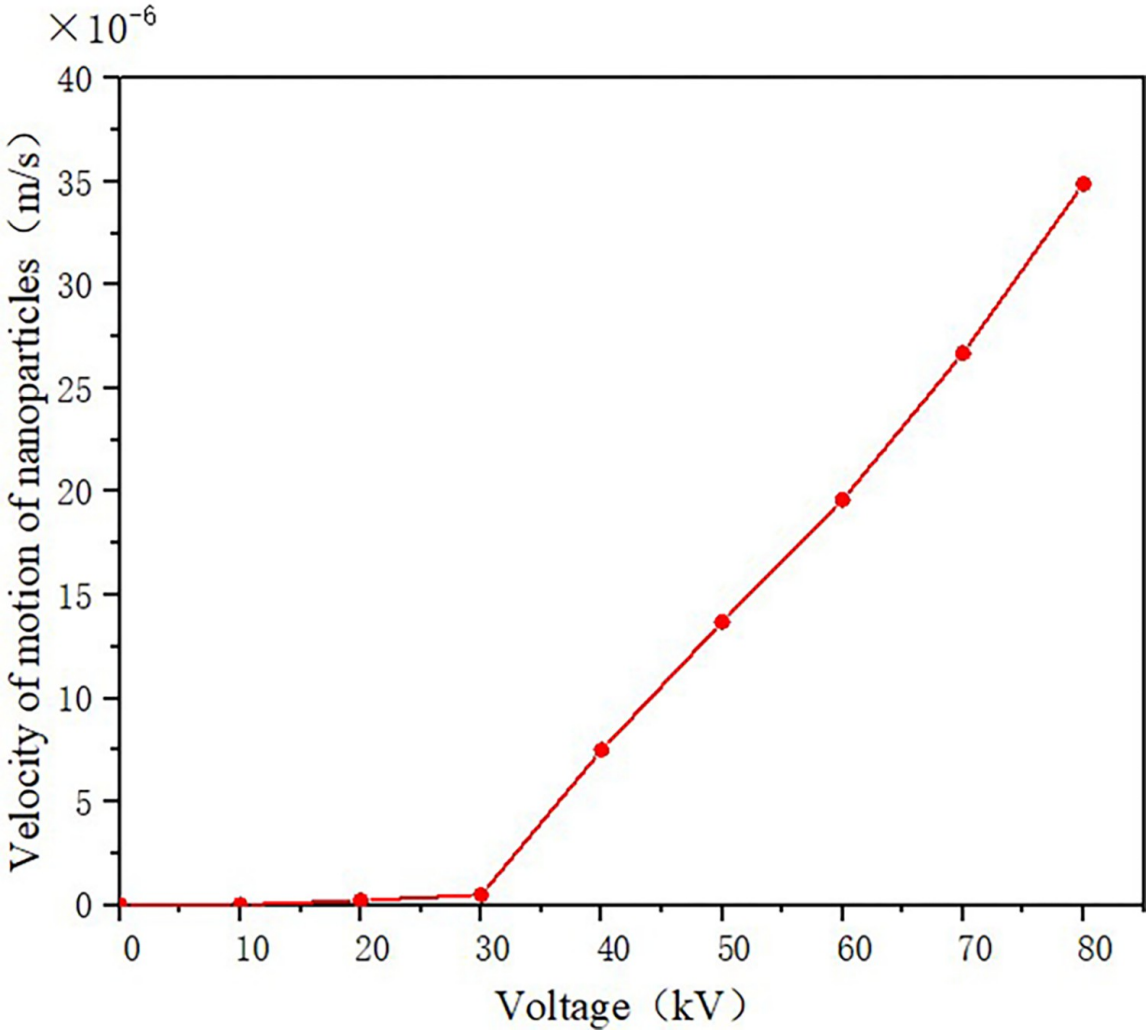
Motion velocity of TiO_2_ nanoparticles.

The relationship between the movement speed of TiO_2_ nanoparticles and the applied voltage is shown in Fig 20. It can be seen that when the applied voltage is low, the movement speed of nanoparticles is negligible, almost close to zero. As the voltage increases to 30kV and above, there is a turning point in the speed, and the speed begins to show a noticeable upward trend. In the range of 30-80kV, the particle’s speed approximately increases linearly with the increasing voltage, reaching its maximum value at 80kV.

**Fig 21:**
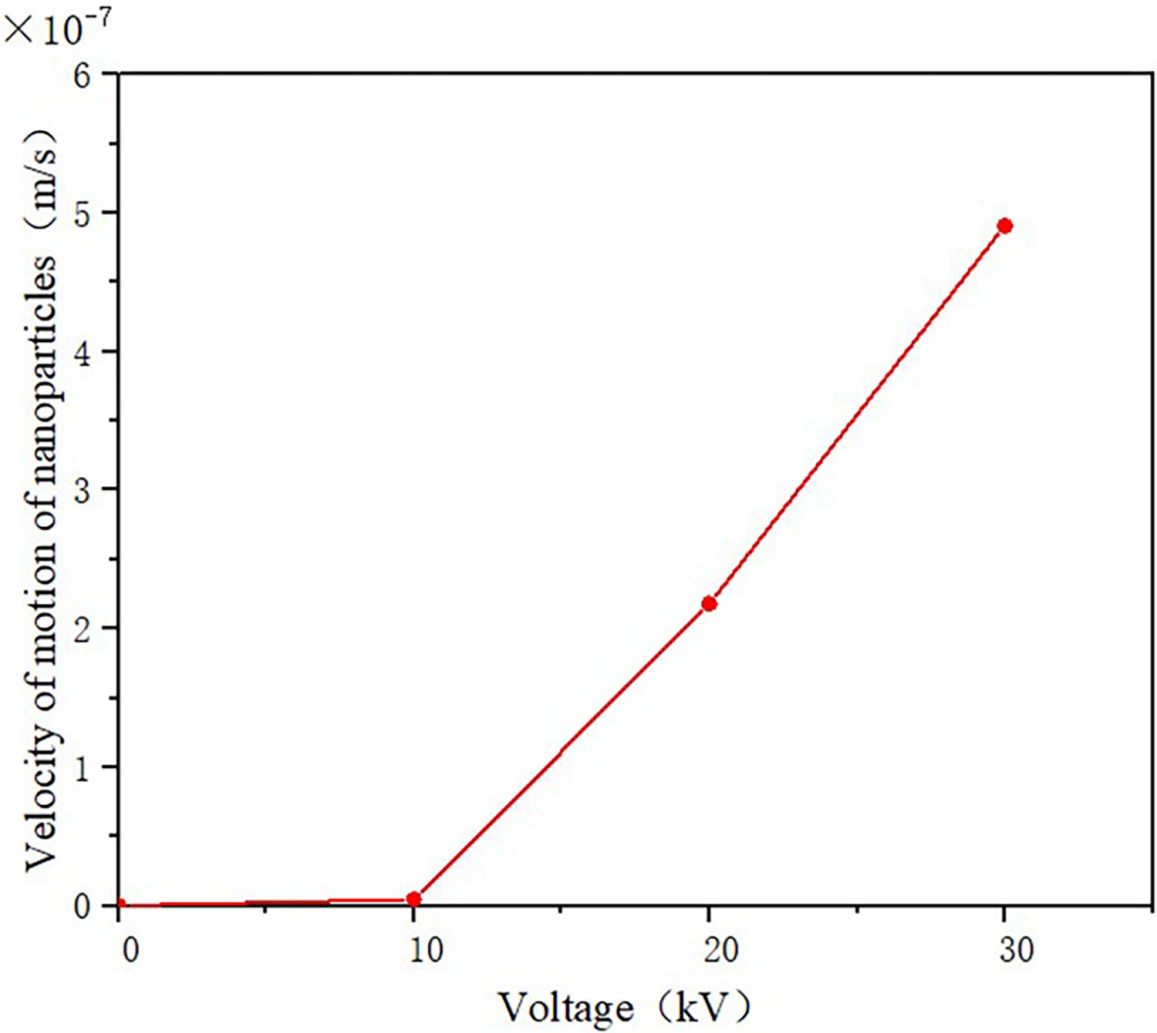
Velocity of TiO_2_ nanoparticles below 70 kV.

To observe the relationship between the movement speed of nanoparticles and the voltage more clearly at low voltages, the speed variation of TiO_2_ nanoparticles under an applied voltage of 0-30kV is plotted in Fig 21. It can be seen that when the voltage is below 10kV, the movement speed of nanoparticles can be neglected. When the applied voltage is between 10-30kV, the movement speed of TiO_2_ nanoparticles shows an approximate linear relationship with the voltage, but with a lower slope.

The reason for the change in the speed of TiO_2_ nanoparticles is that when the applied voltage is low, the number of charges adsorbed by nanoparticles due to polarization is small, resulting in a low charge on the particles and slow motion under the electric field force. With the increase of voltage, the electric field in the oil gap gradually rises, and the number of charges adsorbed by nanoparticles gradually increases, enhancing the negative charge of the particles. Under the action of the electric field, the particles gain a greater speed and move toward the spherical electrode.

Reference [45] was the first to employ the thermal stimulation current (TSC) method to measure the trap characteristics in both pure transformer oil and TiO_2_ nanoparticle-modified insulating oil. The experiment revealed that there was no significant difference in trap depth between the modified and pure oils. However, the trap density in the nanoparticle-modified oil was increased by 85% compared to that in the pure oil, with the trap density in the TiO_2_-modified oil being 1.59 times that of the pure oil. This finding suggests that nanoparticles effectively enhance the trap density in insulating oil.

In the nanofluid, under the polarization effect of TiO_2_ particles, the number of shallow traps in the transformer oil increases, and the trap energy levels are reduced, which is more conducive to the continuous trapping and de-trapping of charges through shallow traps, facilitating their spread in all directions [46]. The increase in the number density of shallow traps affects electron migration in several ways: 1) The probability of electrons being captured by shallow traps increases; 2) The spacing between adjacent traps becomes smaller, making it easier for electrons to hop between them; 3) As the spacing between adjacent shallow traps decreases, the potential barriers between traps become narrower, increasing the probability of electrons tunneling through the traps, further promoting electron migration between traps [47]. After nanofluid adsorbs electrons, due to their much larger particle size and slower movement compared to electrons, the probability of negatively charged nanoparticles undergoing collision ionization in the oil is reduced. Moreover, the recombination between negative ions and positive ions is enhanced, leading to a decrease in space charge and the suppression of streamer development within the oil gap, thereby increasing the breakdown voltage of the oil gap.

**Fig 22:**
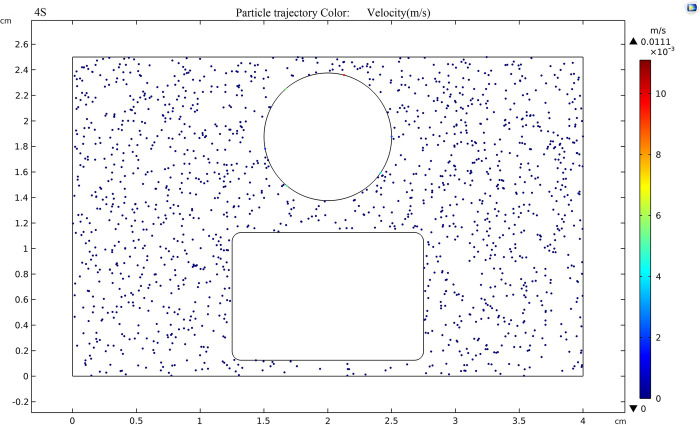
Motion trajectory of nanoparticles under an applied voltage of 80 kV alternating current.

To validate the theoretical basis, this paper conducted a simulation analysis of particle movement speed under an 80kV AC voltage at power frequency, as depicted in Fig 22.

The velocity of TiO_2_ nanoparticles is 1.11 × 10 ^− 5^ m / s, which is one-third the speed under DC voltage, and the nanoparticles show almost no tendency to move towards the electrodes under AC voltage. This suggests that under the influence of AC voltage, TiO_2_ nanoparticles are affected by the changing electric field. The two adjacent polarization directions are opposite, and the direction of acceleration obtained by each polarized particle is also opposite so that the particle velocity can no longer increase steadily in the same direction, resulting in a smaller final velocity of the nanoparticles.

## Conclusion

1. The breakdown voltage of transformer oil decreases as the water content increases. Nanoparticles have a certain effect in enhancing the breakdown voltage of transformer oil across different water levels. At low water content, the higher the breakdown voltage of the pure oil, the more significant the improvement in breakdown voltage caused by the nanoparticles. At medium to high water levels, the enhancement in breakdown voltage of the pure oil by nanoparticles is less pronounced.

2. At higher temperatures, the nanoparticles exhibit a significant enhancing effect on the breakdown voltage of transformer oil. At lower temperatures, the enhancement effect is weaker. Furthermore, at lower temperatures, the resistivity of the nanofluid is lower than that of the pure oil. As the temperature rises, the impact of the nanoparticles on resistivity progressively weakens, and the resistivity of the transformer oil is lower. This is consistent with the experimental results for breakdown voltage.

3. Under the influence of an electric field, nanoparticles undergo polarization, with the positive charges induced on their surfaces capturing high-speed electrons, thereby forming slowly moving negatively charged particles. This causes the centers of positive and negative charges within the streamer to recede from each other at a slower pace, mitigating the distortion of space charge. Moreover, due to the significantly larger diameter of nanoparticles compared to electrons, their movement is also slower, which reduces the probability of collision ionization of negatively charged particles and enhances the recombination of positive and negative charges. Consequently, this serves to suppress the development of the streamer and raises the breakdown voltage.

4. The amount of electronic charge adsorbed by nanoparticles through polarization increases with the increase in electric field strength and particle size. This leads to the acceleration of nanoparticles, which are largely stationary in their initial state, under the influence of the electric field force. At voltages below 10 kV, the movement speed of the nanoparticles is slow, but it increases to some extent as the electric field strength is intensified. When the voltage surpasses 30 kV, there is a significant rise in the speed of the nanoparticles.

## Supporting information

S1 Raw data (XLSX)

## References

[1] Chen YD, Zheng Y, Meng H, Li RZ, Zhang HL, Liang J, et al. Effect of Temperature on Power Frequency Breakdown Characteristics of SF6/CF4 Mixed Gas. High Voltage Engineering. 2021; 47(12): 4169–4176. https://doi.org/DOI:10.13336/j.1003-6520.hve.20201676

[2] Chen YD, Zheng Y, Meng H, Li RZ, Zhang HL, Liang J, et al. Probability Distribution of Insulator Flashover Voltage in Low TemperatureSF6/CF4Gas Mixture. High Voltage Apparatus. 2022; 58(07): 229–236. https://doi.org/DOI:10.13296/j.1001⁃1609.hva.2022.07.028

[3] Xie PB, Shi RX. Study on the compatibility of a nano-TiO2 surface modifier and dispersion solvent. Journal of South China University of Technology (Natural Science Edition). 2023; 51(6). https://doi.org/doi:10.12141/j.issn.1000-565X.220654

[4] Guo HB, Huo XC, Yang Y, Wang Q, Xue SH, Chen TJ. Study on insulation and thermal conductivity of transformer oil modified by rare earth composite particles. Journal of the Chinese Society of Rare Earths. 2023; 1–11.

[5] Zhao MQ, Wang Y, Li JF, Zhang QK, Li HQ, Zhu QD. Molecular simulation on the effect of silane coupling agent modified TiO2 on water molecular diffusion behavior in vegetable insulating oil. Insulating Materials. 2023; 56(11): 57–64. https://doi.org/DOI:10.16790/j.cnki.1009-9239.im.2023.11.010

[6] Li JF, Wang X, Wang Y, Li HQ, Zhu QD. Effect of Silane Coupling Agent Modified TiO2 on the Diffusion Behavior of Water Molecules in Cellulose. Shandong Electric Power. 2023; 50(11): 1–10. https://doi.org/DOI:10.20097/j.cnki.issn1007-9904.2023.11.001

[7] Beheshti A, Shanbedi M, Zeinali Heris S. Heat transfer and rheological properties of transformer oil-oxidized MWCNT nanofluid. Journal of Thermal Analysis and Calorimetry. 2014; 118: 1451–1460. https://doi.org/DOI10.1007/s10973-014-4048-0

[8] Naddaf A, Zeinali Heris S. Experimental study on thermal conductivity and electrical conductivity of diesel oil-based nanofluids of graphene nanoplatelets and carbon nanotubes. International Communications in Heat and Mass Transfer. 2018; 95: 116–122. 10.1016/j.icheatmasstransfer.2018.05.004

[9] Pourpasha H, Zeinali Heris S, Borhan Mousavi S. Thermal performance of novel ZnFe2O4 and TiO_2_-doped MWCNT nanocomposites in transformer oil. Journal of Molecular Liquids. 2024; 394: 123727. 10.1016/j.molliq.2023.123727

[10] Pourpasha H, Zeinali Heris S, Mohammadpourfard M. The effect of TiO2 doped multi-walled carbon nanotubes synthesis on the thermophysical and heat transfer properties of transformer oil: A comprehensive experimental study. Case Studies in Thermal Engineering. 2023; 41: 102607. 10.1016/j.csite.2022.102607

[11] Shanbedi M, Zeinali Heris S, Amiri A, Eshghi H. Synthesis of water-soluble Fe-decorated multi-walled carbon nanotubes: a study on thermo-physical properties of ferromagnetic nanofluid. Journal of the Taiwan Institute of Chemical Engineers. 2016; 60: 547–554. 10.1016/j.jtice.2015.10.008

[12] Alizadeh H, Pourpasha H, Zeinali Heris S, Estelle P. Experimental investigation on the thermal performance of covalently functionalized hydroxylated and non-covalently functionalized multi-walled carbon nanotubes/transformer oil nanofluid. Case Studies in Thermal Engineering. 2022; 31: 101713. 10.1016/j.csite.2021.101713

[13] Niu TX. Study of Physicochemical and Electrical Properties of Insulating Oil Paper Modified by Nano-TiO_2_ under Thermal Aging. Jiangxi University of Science and Technology. 2023. https://doi.org/DOI:10.27176/d.cnki.gnfyc.2023.000169

[14] Guo ZY. Electrical and Thermal Aging Characteristics of Cellulose Insulating Pressboard Modified by Nano-TiO_2_. Jiangxi University of Science and Technology. 2021. https://doi.org/DOI:10.27176/d.cnki.gnfyc.2021.000476

[15] Zhou CH. Study on Space Charge and Breakdown Characteristics of Polyimide Films Modified by Nano Particles Under Strong Electric Field. Jiangxi University of Science and Technology. 2023. https://doi.org/DOI:10.27176/d.cnki.gnfyc.2023.000167

[16] Liu DS, Zhou CH, Ding J, Ye J, Du BX. Review of Research on Nanomodified Oil Paper Composite Insulation for Transformers. Transaction of China Electrotechnical Society. 2023; 38(09): 2464–2479. https://doi.org/DOI:10.19595/j.cnki.1000-6753.tces.221187

[17] Hwang JG, Zahn A, O’Sullivan FM, Pettersson LAA, Hjortstam O, Liu RS. Effects of nanoparticle charging on streamer development in transformer oil-based nanofluids. Journal of Applied Physics. 2010; 107(1): 1–17. doi: 10.1063/1.3267474

[18] Jin HF, Peter M. Partial discharge behavior of mineral oil-based nanofluids. IEEE Transactions on Dielectrics and Electrical Insulation. 2015; 22(5): 2747–2753. https://doi.org/DOI10.1109/TDEI.2015.005145

[19] Ren H, Kou XS, Ma L, Zhao L, Zhou YX, Wang Q. Experimental Study on Application of the Transformer with High⁃thermal⁃conductivity Oil Added by Nano⁃AlN Ceramic Particles. High Voltage Apparatus. 2020; 56(02): 93–100. https://doi.org/DOI:10.13296/j.1001⁃1609.hva.2020.02.014

[20] Shi J, Luo B, Sima WX, Cai HS. Experiment and Simulation of the Effect of Nanoparticles on Oil-Paper Composite Insulation System. Southern Power System Technology. 2015; 9(03): 51–56. https://doi.org/DOI:10.13648/j.cnki.issn1674-0629.2015.03.010

[21] Sima WX, Cao XF, Yang Q, Yu W, Shi J, Song H. Comparison and Analysis on the Impulse Breakdown Characteristics of Three Transformer Oil-based Nanofluids. High Voltage Engineering. 2015; 41(02): 374–381. https://doi.org/DOI:10.13336/j.1003-6520.hve.2015.02.003

[22] Shan BL, Ying YP, Meng H, Niu MK, Li CG. Effect of TiO_2_ Nanoparticles on DC Breakdown Performance of Transformer Oil-Impregnated Pressboard transformer oil-impregnated pressboard. IEEE Transactions on Dielectrics and Electrical Insulation. 2019; 26: 1998–2004. https://doi.org/DOI:10.1109/TDEI.2019.008300

[23] Fernández I, Valiente R, Ortiz F, Renedo C, Ortiz A. Effect of TiO2 and nanoparticles on the performance of dielectric nanofluids based on vegetable esters during their aging. Nanoparticles. 2020; 10(4): 692. https://doi.org/doi:10.3390/nano10040692 10.3390/nano10040692 PMC7221705 32268581

[24] Qin CX, Huang YX, Liu LQ, Liang HJ, Shang JF, Xue YP. Study on Power Frequency Breakdown Characteristics of Nano-TiO2 Modified Transformer Oil under Severe Cold Conditions. Applied Sciences-Basel. 2023; 13(17): 9656. 10.3390/app13179656

[25] Niu MK, Liu BX, Wu YY, Song HL, Huang M, Lv YZ. Characteristics and Mechanism of Corona Discharge in Transformer Oil Modified by TiO_2_ Nanoparticles Under DC Voltage. High Voltage Engineering. 2021; 47(03): 1037–1045. https://doi.org/DOI:10.13336/j.1003-6520.hve.20200377

[26] Wang HJ, Du Q, Zheng TQ, Li Q, Wang L. Influence of Nanoparticles on Ion Migration of Transformer Oil. Insulating Materials. 2019; 52(11): 44–48. https://doi.org/DOI:10.16790/j.cnki.1009-9239.im.2019.11.008

[27] Ge Y. Research on the Charge Transport Mechanism in Transformer oil-based Nanofluid under Impulse Voltage. North China Electric Power University. 2019. https://doi.org/DOI:10.27140/d.cnki.ghbbu.2019.000110.

[28] Miu J, Dong M, Yang YB, Shen LP, Wang H. Modified Electrical Conductivity Model for Transformer Oil-Based Nanofluids. Journal of Xian jiao tong University. 2013; 47(02): 87–91. https://doi.org/DOI:10.7652/xjtuxb201302015

[29] Wang L, Niu JM, Li C, Lv YZ, Huang M, Li CR. Microscopic Model of Breakdown and Conduction in TiO_2_ Nanoparticles Modified Transformer Oil. High Voltage Engineering. 2019; 45(10): 3350–3356. https://doi.org/DOI:10.13336/j.1003-6520.hve.20180109

[30] Farade R, Wahab N. The Effect of Interfacial Zone Due to Nanoparticle–Surfactant Interaction on Dielectric Properties of Vegetable Oil Based Nanofluids. IEEE Access. 2021; 9: 107033–107045. https://doi.org/DOI:10.1109/ACCESS.2021.3098758

[31] Yang T, Wang FP, Yao DG, Li J, Zheng HB, Yao W, et al. Low-Temperature Property Improvement on Green and Low-Carbon Natural Ester Insulating Oil. IEEE Transactions on Dielectrics and Electrical Insulation. 2022; 29(4): 1459–1464. https://doi.org/DOI:10.1109/TDEI.2022.3179224

[32] Daghrah M, Wang ZD, Liu Q, Hilker A, Gyore A. Experimental Study of the Influence of Different Liquids on the Transformer Cooling Performance. IEEE Transactions on Power Delivery. 2019; 34(2): 588–595. https://doi.org/DOI:10.1109/TPWRD.2019.2895533

[33] Fan BT, Ji SC, Shao MY, Bu ZW. Effects of AC Voltage Amplitude and Frequency on Partial Discharge Characteristics and Insulation Life of Oil-Paper System Tip Defects. Transformer. 2023; 60(11): 38–45. https://doi.org/DOI:10.19487/j.cnki.1001-8425.2023.11.004

[34] Hu XC, Xue SH, Sun LQ, Wu LX, Liu J, Song ZP. Effect of antioxidant types in transformer oil on thermal aging performance of insulating paper. Insulating Materials. 2023; 56(11): 47–52. https://doi.org/DOI:10.16790/j.cnki.1009-9239.im.2023.11.008

[35] Khaled U, Beroual A. AC Dielectric Strength of Synthetic Ester-Based Fe3O4, Al2O3 and SiO2 Nanofluids–Conformity with Normal and Weibull Distributions. IEEE Transactions on Dielectrics and Electrical Insulation. 2019; 26(2): 625–633. https://doi.org/DOI:10.1109/TDEI.2018.007759

[36] Khaled U, Beroual A. Statistical Investigation of AC Dielectric Strength of Natural Ester Oil-Based Fe3O4, Al2O3, and SiO2Nano-Fluids. IEEE Access. 2019; 7. 60594–60601. https://doi.org/DOI:10.1109/ACCESS.2019.2915517

[37] Zhou YX, Kou XS, Yang Y, Song W, Liu DL, Ren H, et al. Research Status on Synthesis and Insulating Properties of Nano-modified Transformer Oil. Insulating Materials. 2016; 49(11): 26–35. https://doi.org/DOI:10.16790/j.cnki.1009-9239.im.2016.11.005

[38] Chen QL. The Influence Mechanism of Nanoparticle Charging-Discharging Behavior and Microscopic Disturbance on Streamer Evolution in Insulating Oil. Chongqing University. 2021. https://doi.org/DOI:10.27670/d.cnki.gcqdu.2021.004270.

[39] Zhou Y, Cui W, Zhang JJ, Zhou DM, Lv YZ, Li CR. Measurements and Mechanism Analysis of Propagating Characteristics of Positive Streamer in TiO_2_ Transformer Oil Based Nanofluids. High Voltage Engineering. 2018; 44(09): 2947–2952. https://doi.org/DOI:10.13336/j.1003-6520.hve.20180828024

[40] Hong ZH, Yan B, Qian GC, Zou DX, Zhang R, Zhao JN. Cause Analysis of a 110kV Oil-oil Oil Paper Insulation Transformer Bushing Fault. Transformer. 2021; 58(12): 72–77. https://doi.org/DOI:10.19487/j.cnki.1001-8425.2021.12.015

[41] Liu YP, Zhao JY, Liu HC, Zhao T, Yi ZA. Insulation Characteristics and Influencing Factors of Transformer Oil Containing Cellulose Particles under Low-Frequency Voltage. Transactions of China Electrotechnical Society. 2024; 39(04): 1198–1207. doi: 10.19595/j.cnki.1000-6753.tces.222061

[42] Li XH, Zhang BZ, Duan JP, Wang JY, Qu Z, Ji MM. Particle continuous separation simulation based on 3D electrode coupled with dielectric electrophoresis force and inertia force. Transducer and Microsystem Technologies. 2022; 41(03): 43–46. https://doi.org/DOI:10.13873/J.1000—9787(2022)03—0043—04

[43] Zhang GZ, Yan WY, Wang K, Chen K, Zhang XX. Simulation Study on Motion Characteristics of Fiber Impurity Particles in Flowing Insulating Oil. Transactions of China Electrotechnical Society. 2023; 38(09): 2500–2509. https://doi.org/DOI:10.19595/j.cnki.1000-6753.tces.211957

[44] Shi J. Study on Characteristics of Space Charge Distribution and Streamer Discharge in Transformer Oil Based Nanofluids Under Switching Impulse Voltage. Chongqing University. 2014.

[45] Du YF, Lv YZ, Li CR, Chen MT, Zhong YX, Zhou JQ, et al. Effect of semiconductive nanoparticles on insulating performances of transformer oil. IEEE Transactions on Dielectrics and Electrical Insulation. 2012; 19(3): 770–776. https://doi.org/DOI:10.1109/TDEI.2012.6215079

[46] Wang L, Niu MK, Ying YP, Lv YZ, Li CR. Streamer Characteristics of TiO2 Nanofluid/Pressboard System with Different Nanoparticle Size. Transactions of China Electrotechnical Society. 2019; 34(07): 1544–1552. https://doi.org/DOI:10.19595/j.cnki.1000-6753.tces.180430

[47] Huang M, Niu MK, Ying YP, Lv YZ, Ge Y, Li CR. Properties and Mechanism of Electron Transfer in Transformer Oil Modified with TiO_2_ Nanoparticles. High Voltage Engineering. 2020; 46(12): 4220–4226. https://doi.org/DOI:10.13336/j.1003-6520.hve.20190902

